# Lapatinib-Loaded ZIF‑8 Nanoparticles: A Multifunctional Drug Delivery System with Anticancer, Antibacterial, and Antioxidant Properties

**DOI:** 10.1021/acsomega.5c03165

**Published:** 2025-11-28

**Authors:** Ezgi Aslan, Gülşah Şanlı-Mohamed

**Affiliations:** Department of Chemistry, 52972 İzmir Institute of Technology, İzmir 35430, Türkiye

## Abstract

The pitfalls of conventional chemotherapy, including poor solubility, off-target toxicity, and multidrug resistance, have driven the development of nanoparticle-based delivery systems. Here, we report the facile one-pot synthesis of lapatinib-encapsulated zeolitic imidazolate framework-8 (LAP@ZIF-8) nanoparticles. The formulation achieved an encapsulation efficiency of 72.4% and a drug loading capacity of 6.6%. Comprehensive physicochemical characterization confirmed uniform hexagonal morphology (SEM), favorable hydrodynamic size (236 ± 2 nm; DLS), positive surface charge (+29 mV; ζ-potential), high crystallinity (XRD), and excellent thermal stability (TGA). LAP release was pH-responsive, with ∼77% cumulative release at pH 5.5 (tumor-mimicking) versus 43% at pH 7.4 after 96 h. Serum–protein binding (<11%) and hemolysis (<2%) assays demonstrated good biocompatibility. *In vitro*, LAP@ZIF-8 exhibited potent, selective cytotoxicity toward HER2-positive SK-BR-3 breast-cancer cells (72 h IC_50_ = 1.2 μg mL^–1^) while sparing HER2-negative MCF-7 cells. Importantly, both free LAP and LAP@ZIF-8 were well-tolerated by nontumorigenic MCF-10A mammary epithelial cells: viability remained ≥90% at ≤1 μg mL^–1^ and exceeded 50% even at 100 μg mL^–1^, indicating that the IC_50_ was not reached and providing a preliminary safety window for healthy tissues. Beyond its anticancer effects, the nanocarrier displayed broad-spectrum antibacterial activity (minimum bactericidal concentrations: 5 mg mL^–1^ for *Staphylococcus aureus* and 10 mg mL^–1^ for *Escherichia coli*) and moderate antioxidant capacity (DPPH IC_50_ = 666 μg mL^–1^). Collectively, these results position LAP@ZIF-8 as a versatile, pH-sensitive platform that combines selective anticancer efficacy with low toxicity to healthy cells alongside ancillary antibacterial and antioxidant properties suitable for multimodal therapy.

## Graphical abstract

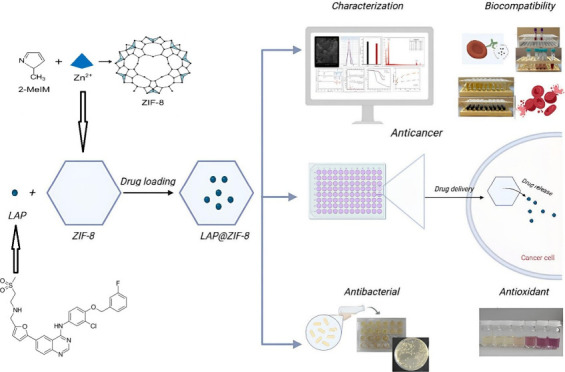

## 1 Introduction

Cancer remains a significant global health issue, with its prevalence and associated mortality rates increasing over time. Breast cancer, which accounted for 11.7% of all cancer cases worldwide in 2020,^1^ became the second most common cancer globally in 2022, representing 11.6% of cases. Among women, breast cancer remains the most frequently diagnosed malignancy.^2^ The overexpression of human epidermal growth factor receptor 2 (HER2), an oncogene that regulates cell growth and differentiation,^3^ is observed in approximately 20–25% of breast cancer cases and is associated with aggressive disease progression.^4^

Lapatinib (LAP; GW 572016; Tykerb, GlaxoSmithKline), the first dual inhibitor targeting the epidermal growth factor receptor (EGFR/HER1) and HER2/erythroblastic leukemia viral oncogene homologue 2 (ErbB2) tyrosine kinases was approved by the U.S. Food and Drug Administration (FDA) in 2007.^5^ LAP, a hydrophobic compound with low water solubility (approximately 0.007 mg/mL), is derived from the quinazoline core structure.^6^ Despite its therapeutic effectiveness, LAP’s poor water solubility significantly reduces its intestinal absorption and bioavailability while also causing damage to the gastrointestinal tract, limiting its use as an injectable drug. This challenge has underscored the need for nanoparticle-based delivery systems to enhance its solubility and therapeutic efficacy.^7^ Moreover, most anticancer drugs lack specificity between cancerous and normal cells, leading to systemic toxicity and adverse effects.^8^ The limitations of current cancer therapies, including severe side effects and drug resistance, have necessitated the development of novel therapeutic strategies and advanced drug delivery systems.^9^ To address all of these challenges, nanoparticle-based drug delivery systems have emerged as promising platforms, offering improved solubility, targeted delivery, and reduced systemic toxicity.

Nanotechnology has recently garnered increasing attention for its role in the diagnosis and treatment of tumors.^10^ Nanoparticles used in cancer treatment offer several advantages, such as addressing the problem of poor solubility by enhancing the bioavailability of the loaded drug, enabling slow release, and improving drug permeability to cancer cells.^11^ Nanoscale MOFs have emerged as promising candidates in drug delivery systems due to their flexible composition, large surface area, degradability, and versatile surface properties.^12^ Among MOFs, zeolitic imidazolate frameworks (ZIFs) are particularly well-studied for drug delivery applications, owing to their biocompatibility at low concentrations, ease of synthesis, and pH-responsive properties.^13^ Zeolitic imidazolate framework-8 (ZIF-8), a prominent subgroup of ZIFs, is formed through the coordination of zinc ions (Zn^2+^) and nitrogen atoms on the 2-methylimidazole (2-MeIm) ring. While metal–organic frameworks do not possess inherent anticancer properties, they are widely employed as nanocarriers that enhance the efficacy, solubility, and selectivity of encapsulated anticancer agents through controlled and targeted delivery mechanisms.^14^ In addition to their role as anticancer drug carriers, certain MOFs have demonstrated potential antimicrobial activity, making them promising candidates for use as antibiotic alternatives or supplements in nanomedicine applications.^15^ ZIF-8 possesses several notable features, including high porosity, ease of modification, significant thermal and chemical stability, low toxicity, and excellent biocompatibility.^16^

Numerous studies have demonstrated the successful encapsulation of various anticancer agents into the ZIF-8 frameworks. For example, 5-fluorouracil (5-Fu) was among the first chemotherapeutics incorporated into ZIF-8, where pH-triggered release significantly enhanced drug availability and therapeutic efficacy.^17^ Camptothecin (CPT), a hydrophobic topoisomerase inhibitor, has also been loaded into ZIF-8 to improve its solubility and sustained release.^18^ Doxorubicin (DOX), perhaps the most commonly studied agent in MOF-based systems, has been encapsulated in ZIF-8 to reduce cardiotoxicity while maintaining anticancer potency.^19^ Similarly, 6-mercaptopurine (6-MP) has been formulated with ZIF-8 to improve its pharmacokinetic profile and minimize degradation.^20^

Various formulations for LAP delivery have also been developed, including polymer–lipid hybrid nanoparticles, lyophilized polymeric micelles, exosomes, and gold nanorods, each demonstrating promising anticancer effects in HER2-positive breast cancer models. For instance, LAP-loaded polymer–lipid hybrid nanoparticles improved drug solubility and cellular uptake but lacked tunable release and multifunctionality.^21^ Polymeric micelle-based LAP formulations achieved moderate stability and tumor accumulation; however, their long-term systemic safety and additional therapeutic functions (e.g., antimicrobial or antioxidant activity) were not addressed.^22^ Exosome-based LAP delivery systems exhibited excellent biocompatibility and cell-specific uptake, yet they suffer from limited scalability and structural uniformity challenges.^23^ LAP-loaded gold nanorods demonstrated photothermal-enhanced drug release but require external stimuli (e.g., NIR light) and pose potential concerns related to metal accumulation and toxicity.^24^

In addition to serving as carriers for anticancer agents, MOFs have shown promise as supplements or alternatives to antibiotics due to their unique structural features and metal ion components. The antibacterial effects of MOFs are often attributed to physical damage to bacterial cells, which may result either from guest molecules incorporated into their cavities or from the properties of the metal components within the framework.^25^ The literature reports that Zn^2+^ induces bacterial death by interfering with intracellular biochemical pathways, including the production of reactive oxygen species (ROS) and disruption of cell cycle mechanisms. The antibacterial effect of ZIF-8 arises from both the Zn^2+^ ions and the organic 2-methylimidazole (2-MeIm) ligand, which facilitates interactions with bacterial cell walls.^26^ However, Zn^2+^ requires a long time to exert its antibacterial effects, which may contribute to the development of zinc resistance in bacteria.^27^ The increasing use of antibiotics has led to the emergence of multidrug-resistant bacteria, prompting the search for alternative drugs with fewer or no side effects.^28^ In a study involving the anticancer drug doxorubicin (DOX), it was demonstrated that the synthesized DOX@ZIF-8 exhibited a strong inhibition zone against Gram-negative bacteria, specifically *Escherichia coli* (*E. coli*).^29^ Based on this finding, it was hypothesized that the combination of LAP and ZIF-8 as an antibacterial strategy could provide enhanced antibacterial activity through a synergistic effect, surpassing the efficacy of the drug or MOF alone.

Additionally, MOFs and nanoparticles have been explored for various antioxidant applications due to their unique properties.^30^ Free radicals, harmful molecules produced in biological systems, can cause significant cellular damage and various disorders. Antioxidants are compounds that neutralize free radicals by intervening in oxidative processes within living tissues, thereby mitigating damage caused by the formation of reactive oxygen species. They achieve this by undergoing oxidation themselves.^31^ In the same study involving DOX, it was also reported that the synthesized DOX@ZIF-8 exhibited high antioxidant activity.^29^

This study explores the potential of a ZIF-8-based drug delivery system as a multifunctional agent with cytotoxic, antibacterial, and antioxidant properties. LAP, a clinically approved dual-HER2/EGFR tyrosine-kinase inhibitor for breast cancer, offers significant therapeutic advantages, but its clinical utility is hampered by poor solubility and systemic side effects. Encapsulating LAP in metal–organic frameworks (MOFs) such as ZIF-8 can provide controlled, pH-responsive release and mitigate off-target toxicity. While previous studies have encapsulated various chemotherapeutics (e.g., doxorubicin, camptothecin, and 5-fluorouracil) into ZIF-8 for cancer therapy and alternative LAP formulations have included liposomes, polymeric micelles, exosomes, and gold nanorods, no prior report has described the formulation and evaluation of LAP-loaded ZIF-8 nanoparticles. The combined cytotoxic, antibacterial, and antioxidant activities of LAP@ZIF-8 have not been comprehensively investigated, representing a critical gap in the literature.

Here, we introduce a one-pot synthesis of LAP@ZIF-8 nanoparticles and evaluate their physicochemical and biological performance. In addition to HER2-positive SKBR-3 and HER2-negative MCF-7 breast-cancer cells, we include nontumorigenic human mammary epithelial MCF-10A cells as a healthy counterpart to rigorously assess biocompatibility and cancer selectivity. Our primary objective is to develop a biocompatible, biodegradable, pH-sensitive nanocarrier with enhanced cytotoxicity toward breast-cancer cells while sparing normal tissues. Cytotoxicity assays reveal that LAP@ZIF-8 markedly suppresses SKBR-3 and MCF-7 viability yet maintains >50% viability in MCF-10A cells even at the highest concentration tested, underscoring its therapeutic selectivity. The nanocarrier’s antibacterial activity is demonstrated against Gram-negative (*Escherichia coli*) and Gram-positive (*Staphylococcus aureus* (*S. aureus*)) strains, and its antioxidant potential is confirmed via DPPH free radical scavenging.

In our study, LAP@ZIF-8 is presented as the first example of this formulation, providing a unique pH-sensitive delivery mechanism with added antibacterial and antioxidant functionalities. In contrast to existing LAP formulations, which primarily focus on anticancer effects, we conduct a comprehensive analysis including HER2-targeted cytotoxicity, hemocompatibility, serum–protein interaction, antibacterial efficacy, and antioxidant activity. These combined evaluations, along with comparisons to existing drug@ZIF-8 systems and non-MOF LAP carriers, distinguish our work and highlight the multifunctional therapeutic potential of LAP@ZIF-8 as a versatile nanoplatform.

## 2 Materials and Methods

### 2.1 Materials

All chemicals and reagents were obtained from Sigma-Aldrich (Merck, Darmstadt, Germany) unless otherwise specified and were used without further purification. Dimethyl sulfoxide (DMSO, cat. no. D2650, ≥99.9%), zinc nitrate hexahydrate [Zn­(NO_3_)_2_·6H_2_O, cat. no. 228737, ≥98%], and 2-methylimidazole (2-MeIm, cat. no. 693651, ≥99%) were employed for the synthesis of ZIF-8 and drug-loaded formulations. Lapatinib ditosylate (cat. no. S1028, Selleckchem, ≥98% purity by HPLC) was used as the model drug. Ethanol (cat. no. 24102, 30% v/v aqueous solution) and phosphate-buffered saline (PBS, cat. no. P3813, pH 7.4) were used for washing and release media. Triton X-100 (catalog no. T8787) was used as the positive control in hemolysis studies. The DPPH radical (catalog no. 70900, Cayman Chemical, ≥95%) and ascorbic acid (catalog no. A92902, ≥99%) were used in antioxidant assays. Fetal bovine serum (FBS, cat. no. 10270106, Gibco, heat-inactivated) and MTT reagent (cat. no. MTT-500, GoldBio, cell culture grade) were used in biocompatibility and cytotoxicity experiments. Protein quantification was performed using a BCA protein assay kit (cat. no. 23225, Thermo Fisher Scientific). A dialysis membrane with a molecular weight cutoff (MWCO) of 14 kDa (cat. no. TX0111, BioBasic) was employed for drug release studies.

### 2.2 Methods

#### 2.2.1 Synthesis of ZIF-8 and LAP@ZIF-8

ZIF-8 (C8H10N4Zn) nanoparticles were synthesized via a one-pot method using a Zn^2+^:2-MeIm:H_2_O molar ratio of 1:70:1238.^20,32^ Specifically, 58.5 mg of zinc nitrate hexahydrate [Zn­(NO_3_)_2_·6H_2_O] was dissolved in 0.4 mL of deionized water to prepare the metal precursor solution. Separately, 1135 mg of 2-methylimidazole (2-MeIm) was dissolved in 4 mL of deionized water, and 0.6 mL of DMSO was added to facilitate solubility and dispersion. The zinc nitrate solution was then added dropwise to the 2-MeIm/DMSO solution under constant stirring at room temperature (25 °C). A milky white suspension formed immediately and was stirred for 15 min. The resulting dispersion was centrifuged at 13,500 rpm for 15 min, and the solid was washed three times with 30% ethanol in water to remove unreacted precursors. The purified product was dried in a vacuum oven at 60 °C for 12 h, yielding dry ZIF-8 nanoparticles as a white powder.

For the synthesis of LAP-loaded ZIF-8 (LAP@ZIF-8), 3 mg of LAP ditosylate was first dissolved in 0.6 mL of DMSO and added to the 2-MeIm solution before the addition of the zinc nitrate solution. The rest of the synthesis followed the same procedure as that described above for ZIF-8. After purification and drying, the final LAP@ZIF-8 product appeared as a light-yellow powder, indicating successful drug incorporation.

To evaluate batch-to-batch reproducibility and formulation quality, we conducted triplicate syntheses of both ZIF-8 and LAP@ZIF-8 nanoparticles under identical conditions.

##### 2.2.1.1 The Synthesis Yield of ZIF-8

The yield of ZIF-8 was defined as the ratio of the amount of solid products obtained from the synthesis mixture to the maximum possible amount of ZIF-8 that can be produced from the synthesis mixture if all limiting reactant was consumed. The synthesis yield value of the ZIF-8 was calculated using the amount obtained as a result of the synthesis and the following formation reaction of ZIF-8:

$$\mathrm{Zn}{\left({\mathrm{NO}}_{3}\right)}_{2}\cdot 6H_{2}O+C_{4}H_{6}N_{2}\to C_{8}H_{10}N_{4}\mathrm{Zn}$$

#### 2.2.2 Characterization of ZIF-8 and LAP@ZIF-8

##### 2.2.2.1 Encapsulation Efficiency and Drug Loading

The nanoparticles were synthesized, and encapsulation efficiency and drug loading were determined by the gravimetric method^33^ using a UV–vis spectrometer (Shimadzu-UV-2550, Japan and PerkinElmer LAMBDA Bio+). Supernatants were collected during the washing step of the synthesis of LAP@ZIF-8. To determine the drug loading efficiency, the supernatant was analyzed by a UV–vis spectrometer at a wavelength of 270 nm. The amount of LAP encapsulated into the nanoparticle was determined by an indirect method. Free LAP was determined by the UV–vis spectrophotometric method in the supernatant after three wash cycles. Supernatant concentration calculation was calculated using eqs 1 and 2:

$$A_{\mathrm{meas}}=0.0226\cdot C_{\mathrm{meas}}-0.0459$$ 1

$$C_{\mathrm{supernatant}}\times V_{\mathrm{supernatant}}=C_{\mathrm{meas}}\times V_{\mathrm{DMSO}}$$ 2

where *A*_meas_ is the measured absorbance, *C*_meas_ is the corresponding concentration of free LAP, *C*_supernatant_ is the supernatant concentration, *V*_supernatant_ is the volume of the LAP supernatant taken, and *V*_DMSO_ is the volume of DMSO used to dilute it. The concentration of the prepared solution (*C*_prepared_) was calculated using eq 3:

$$C_{\mathrm{stock}}\times V_{\mathrm{stock}}=C_{\mathrm{prepared}}\times V_{\mathrm{tot}}$$ 3

where *C*_stock_ is the concentration of the LAP stock solution (mg/mL), *V*_stock_ is the volume of LAP stock solution taken for synthesis, and *V*_tot_ is the total synthesis volume. The amount of LAP encapsulated (*C*_encapsulated_) was calculated using eq 4:

$$C_{\mathrm{encapsulated}}=C_{\mathrm{prepared}}-C_{\mathrm{supernatant}}$$ 4

The encapsulation efficiency (EE) and drug loading percentage (%DL) were calculated using eqs 5 and 6, respectively:

$$\mathrm{EE}=\frac{C_{\mathrm{encapsulated}}}{C_{\mathrm{prepared}}}\times 100\%$$ 5

$$\%\mathrm{DL}=\frac{C_{\mathrm{encapsulated}}}{C_{\mathrm{NPs}}}\times 100\%$$ 6

where *C*_NPs_ is the total amount of nanoparticles.

##### 2.2.2.2 Structural Analysis

The shape, size, size distribution, structure and crystallinity, composition, and surface charge of the nanoparticles to be used in the study were analyzed, and their characterization properties were determined. Dynamic light scattering (DLS (DLS nanoparticle size analyzer, Particulate Systems–NanoPlus) analysis is based on the diffusive motion of particles in solution. Zeta potential occurs between the particle and the liquid in which the particle is located. The size distribution and potential of the nanoparticle in the aqueous environment were determined by using a zeta potential analyzer (Particulate Systems-NanoPlus). The dimensions of the nanoparticles were determined by scanning electron microscope (SEM) (FEI QUANTA 250 FEG) measurement. The degree of crystallinity of the nanoparticles was determined by X-ray diffraction-XRD (Philips X’Pert Pro diffractometer, Royal Philips Electronics, Amsterdam, The Netherlands), while the elemental composition and potential impurities were assessed by energy-dispersive X-ray spectroscopy (EDX) (FEI QUANTA 250 FEG). Functional groups in nanoparticles were examined by Fourier-transform infrared (FTIR) (PerkinElmer Spectrum Two FTIR spectrometer) spectroscopy analysis. Thermal stability of nanoparticles was examined by thermogravimetric analysis (TGA) (PerkinElmer Diamond TG/DTA).

##### 2.2.2.3 Drug Release Study

Tumor cells have an acidic pH due to their rapid metabolism and anaerobic respiration. This encourages current drug release kinetics to be controlled at pH 5.5, which mimics the inner environment of tumor cells, where controlled decomposition of ZIF-8 supported by acidic pH liberates LAP. A dialysis membrane (MWCO 14,000 Da, 34 mm, TX0111, BioBasic) was used in the release study. Consequently, the release kinetics were investigated in PBS at pH = 7.4 and 5.5 at physiological temperature (37 °C). First, 6 mg of the LAP@ZIF-8 was dispersed in 2 mL of PBS (pH 5.5 or 7.4) by using a sonicator (Elma Ultrasonic Cleaners Elmasonic S). The solution was transferred into the dialysis membrane and immersed in a container containing 40 mL of pH 5.5 or pH 7.4 of PBS. Then, shaking was performed with a stirrer at 37 °C. Samples were taken at certain time intervals, and LAP concentration was measured at 270 nm (maximum absorbance peak given by the drug) on a UV–vis spectrometer (PerkinElmer LAMBDA Bio+). The cumulative drug release (CDR) of LAP was calculated according to eq 7:^34^

$$\mathrm{CDR}=\frac{1}{M_{0}}\sum_{i=1}^{n}M_{\mathrm{i̇}}\times 100\%$$ 7

where *M*_*i*_ is the amount of LAP released from LAP@ZIF-8 at time *i* and *M*_0_ is the total amount of loaded drug in LAP@ZIF-8.

##### 2.2.2.4 Stability Study of LAP@ZIF-8 Nanoparticles

To evaluate the physicochemical stability of LAP@ZIF-8 nanoparticles over time, samples were stored at 4 °C in sealed microcentrifuge tubes protected from light. Stability was monitored on days 1, 3, 5, 7, 15, and 30. At each time point, triplicate aliquots were withdrawn and analyzed for free drug leakage, hydrodynamic size, polydispersity index (PDI), and zeta potential. Free lapatinib released into the supernatant was quantified by UV–vis spectrophotometry at 270 nm following centrifugation (13,500 rpm, 15 min). The particle size and PDI were measured using dynamic light scattering (DLS), while the zeta potential was determined to assess changes in surface charge and colloidal stability. All measurements were performed in triplicate and reported as the mean ± standard deviation. This protocol was designed to assess drug retention, structural integrity, and formulation stability of LAP@ZIF-8 nanoparticles under refrigerated storage conditions.

##### 2.2.2.5 Biocompatibility Assay: Serum–Protein Binding

Possible binding of samples to serum proteins was evaluated by analysis using a bicinchoninic acid (BCA) protein assay kit (The Pierce, Thermo Fisher Scientific). To determine protein binding rates to the drug carrier system, the method applied by Semete et al.^35^ was modified and used. Protein standards were prepared using the guidance included in the protein assay kit. Absorbance values were read at 595 nm against the blank (distilled water). Fetal bovine serum (FBS, Gibco) samples were prepared at various volumes with a total volume of 1000 μL. Samples were incubated in a 37 °C (body temperature) water bath and 150 rpm for 2 h. After incubation, the samples were centrifuged at 13,500 rpm for 15 min. Reagent A, a carbonate buffer containing BCA reagent, and reagent B, a cupric sulfate solution, were mixed to make a green working solution. After centrifugation, 1800 μL of BCA reagent (reagent A + reagent B) (working solution that will turn purple after 30 min at 37 °C in the presence of protein) was added into 200 μL of samples. Samples containing BCA reagent were mixed in a water bath at 37 °C for 30 min. Absorbance values were read at 595 nm against the blank. Protein determination was performed in the supernatant according to the Bradford method,^36^ and the percentage of binding to serum proteins was calculated based on the remaining unbound protein in the supernatant. Using the prepared BSA standard calibration curve, the amounts of initially added protein and unbound protein were calculated. The amount of unbound protein (*C*_unbound_) was subtracted from the amount of initially added protein (*C*_initial_) to obtain the amount of bound protein. The protein binding percentage (%PB) was calculated using eq 8:

$$\%\mathrm{PB}=\frac{C_{\mathrm{initial}}-C_{\mathrm{unbound}}}{C_{\mathrm{initial}}}\times 100\%$$ 8

##### 2.2.2.6 Biocompatibility Assay: Hemocompatibility (Hemolysis)

The damage caused by nanoparticles on erythrocytes was determined by an *in vitro* hemolysis assay. To determine the amount of hemolysis, the method described by Neun et al.^37^ was used. Hemolysis caused by the samples was evaluated photometrically. Two tubes of blood were collected from human subjects at our health facility into EDTA tubes and centrifuged at 13,500 rpm for 15 min to remove plasma and leukocytes. This process was repeated three times. The pellet consisting of erythrocytes was washed two times with 1× PBS (pH 7.4). Erythrocytes were washed with PBS and suspended in PBS to a final concentration of 2%. Samples of varying concentrations (2 μg/mL, 10 μg/mL vs 20 μg/mL) and erythrocyte suspension were prepared in a 1:1 ratio and incubated at 37 °C and 100 rpm for 4.5 h. As positive (100% hemolysis) and negative (0% hemolysis) hemolysis controls, suspensions of erythrocytes in 1% Triton X-100 (nonionic surfactant, Amresco) and PBS were used, respectively. After incubation, all samples were centrifuged (NÜVE NF 800R) at 4100 rpm for 10 min, and the supernatants were used for measurement. Free hemoglobin in the supernatant was measured photometrically at 540 nm. The hemolysis percentage was calculated using the following eq 9.

$$\%\;\mathrm{hemolysis}=\frac{A_{\mathrm{sample}}-A_{\mathrm{neg\_control}}}{A_{\mathrm{pos\_control}}}\times 100\%$$ 9

where *A*_sample_, *A*_pos_control_, and *A*_neg_control_ are the absorbances of the sample, positive control, and negative control, respectively.

#### 2.2.3 Cell Culture and *In Vitro* Cytotoxicity (MTT) Assay

Human breast cancer cell lines SKBR-3 (HER2^+^, ATCC HTB-30) and MCF-7 (ER^+^, ATCC HTB-22), together with the nontumorigenic mammary epithelial cell line MCF-10A (ATCC CRL-10317), were obtained from the American Type Culture Collection. SKBR-3 cells were propagated in McCoy’s 5A medium (Gibco) supplemented with 10% (v/v) heat-inactivated fetal bovine serum (FBS, Gibco) and 1% penicillin–streptomycin (P/S). MCF-7 cells were cultured in high-glucose Dulbecco’s modified Eagle’s medium (DMEM-HG, Gibco) with the same supplements. MCF-10A cells were maintained in DMEM/F-12 (1:1; Gibco) containing 5% horse serum, 20 ng mL^–1^ epidermal growth factor, 0.5 μg mL^–1^ hydrocortisone, 100 ng mL^–1^ cholera toxin, 10 μg mL^–1^ human insulin, and 1% P/S. Cells were incubated at 37 °C in a humidified 5% CO_2_ atmosphere and used between passages 5–20.

Cells (1 × 10^4^ cells well^–1^ in 100 μL of complete medium) were seeded into clear-flat-bottom 96-well plates and allowed to adhere for 24 h. Serial dilutions of free LAP, blank ZIF-8, and LAP@ZIF-8 (0.001–100 μg mL^–1^, prepared in culture medium) were added in sextuplet (*n* = 6 technical replicates, three independent experiments). Vehicle controls received an equal volume of DMSO (final concentration ≤0.5% v/v), while a 1 μM doxorubicin solution served as a positive cytotoxicity control. Untreated cells were taken as 100% viability controls.

After 24, 48, and 72 h of exposure, 10 μL of MTT reagent (5 mg mL^–1^ in PBS, GoldBio) was added to each well, and the plates were incubated for 4 h at 37 °C. The medium was then removed carefully, the plates were centrifuged (1800 rpm, 10 min), and the purple formazan crystals were solubilized in 100 μL of anhydrous DMSO with orbital shaking at 150 rpm for 15 min. Absorbance was read at 570 nm with a 630 nm reference on a Multiskan GO microplate reader (Thermo Fisher Scientific). Cell viability was calculated according to eq 10:

$$\mathrm{cell}\;\mathrm{viability}=\frac{A_{\mathrm{sample}}-A_{\mathrm{blank}}}{A_{\mathrm{control}}-A_{\mathrm{blank}}}\times 100\%$$ 10

where *A*_sample_, *A*_control_, and *A*_blank_ are the absorbances of the sample, control, and blank, respectively.

IC_50_ values were obtained from nonlinear regression (log­[inhibitor] vs response, variable slope) using GraphPad Prism 10. Results are expressed as means ± SD (*n* = 3).

#### 2.2.4 Antibacterial Activity by the Counting-Colony Method

The antibacterial activity of the LAP and LAP@ZIF-8 nanoparticles was determined using the S. aureus (RSKK 1009, Gram-positive bacteria) and *E. coli* (ATCC 25922, Gram-negative bacteria) bacterial suspension with the counting-colony method according to the ASTM E2149 standard protocol. Pure bacterial cultures were plated on Petri dishes containing tryptic soy agar. Growing bacteria colonies were picked off and mixed with peptone water to adjust concentration to the value of 0.5 in the McFarland (2.5 × 10^7^ colony-forming unit (CFU)/mL for *E. coli*, 3.7 × 10^7^ CFU/mL for S. aureus) standard scale. Next, the bacterial suspension was diluted with tryptic soy broth (the dilution ratio is 1:9) to adjust the concentration to 2.5 × 10^6^ CFU/mL, and this was used as a stock suspension for the antibacterial test. The test materials were sterilized using UV light for 15 min. 990 μL of tryptic soy broth and 10 μL of bacterial suspension were added to the wells of a 24-well plate. It was shaken in a Varioskan at 37 °C for 24 h. Suspensions of bacteria and samples with different dilution ratios (100, 10^–2^, 10^–4^, and 10^–6^) were prepared using peptone water, 100 μL of each was spread on the tryptic soy agar plates and incubated at 37 °C for 24 h, and colonies were counted. The inoculum solution was used as a control. Antibacterial activity, expressed as the percentage of bacterial reduction (% reduction), was calculated using eq 11:^38^

$$\%\;\mathrm{reduction}=\frac{B-A}{B}\times 100$$ 11

where *A* is the concentration (in CFU/mL) for the well containing the treated sample and *B* is the concentration (in CFU/mL) of the growth control or “only inoculum” well.

#### 2.2.5 Antioxidant Activity by a 1,1-Diphenyl-2-picrylhydrazyl (DPPH) Radical Scavenging Assay

The radical scavenging activities of LAP, LAP@ZIF-8, and ascorbic acid (positive control) were tested against the DPPH (Cayman) radical using the method described by Wang et al.,^39^ with a few modifications. First, 3 mL of 0.1 mM DPPH methanolic solution was added to 1 mL of various concentrations (2.5–200 μg/mL) of LAP and LAP@ZIF-8. The mixture was incubated at 25 °C in the dark for 30 min at room temperature. The change in the color (from deep violet to pale yellow) was measured at 517 nm against a blank (pure methanol solvent, Sigma-Aldrich) using a UV–vis spectrophotometer. The initial absorbance of DPPH in methanol was measured at 517 nm and recorded as a control, the ascorbic acid was used as a standard, and the same concentrations were prepared as the sample solutions. The ability to scavenge DPPH radicals, expressed as the radical scavenging activity (RSA), was calculated according to eq 12:

$$\mathrm{RSA}=\frac{A_{\mathrm{control}}-A_{\mathrm{sample}}}{A_{\mathrm{control}}}\times 100\%$$ 12

where *A*_control_ is the absorbance of untreated DPPH/methanol solution and *A*_sample_ is the absorbance of the sample or ascorbic acid-treated DPPH/methanol solution.

#### 2.2.6 Statistical Analysis

Values are expressed as the percentage (%) or mean ± standard deviation of replicates (*n* = 2 or 3). Statistical analysis was done with Excel and GraphPad Prism 9.0.0. MTT analysis results were evaluated by two-tailed paired *t* tests (for two-group comparison) and one-way ANOVA followed by Tukey's multiple comparison test for multiple comparisons (more than two groups). The following thresholds for statistical significance were used: *p* < 0.05 (*), *p* < 0.01 (**), *p* < 0.001 (***), and *p* < 0.0001 (****); ns indicates nonsignificant. A value of *p* < 0.05 was considered statistically significant. In addition, the results were evaluated by calculating the coefficient of variation for the MTT results. The graphs created to visualize results (except MTT results by GraphPad Prism 9.0.0) were drawn using OriginLab Pro 2024 (OriginLab Corporation, USA).

## 3 Results and Discussion

### 3.1 Synthesis of ZIF-8 and LAP@ZIF-8 and Yield of ZIF-8

ZIF-8 and LAP@ZIF-8 were produced by a one-pot method. The yield of ZIF-8 synthesis was calculated as a percentage by considering the amount of the substance synthesized and the synthesis reaction. As a result of the calculation, the yield was determined as 58.52 ± 2.44%.

### 3.2 Characterization of ZIF-8 and LAP@ZIF-8

#### 3.2.1 Encapsulation Efficiency and Drug Loading

The synthesized LAP@ZIF-8 was evaluated for its encapsulation efficiency and drug loading capacity. The results demonstrated high values for both parameters. Specifically, the LAP@ZIF-8 formulation, prepared using 3 mg of LAP, exhibited an encapsulation efficiency of 72.42% and a drug loading of 6.55%. This corresponds to the incorporation of 2.173 mg of LAP within the LAP@ZIF-8 nanoparticles. The observed drug loading capacity of 6.55% may appear to be modest. This value may largely have been influenced by the high porosity but limited hydrophobic cavity volume of ZIF-8 and the relatively large molecular size and hydrophobic nature of LAP, which can restrict loading efficiency due to steric hindrance and solubility constraints during encapsulation. Despite the moderate DL%, the encapsulation efficiency (72.42%) was high, suggesting that most of the drug added was successfully incorporated, albeit in limited total quantity due to capacity constraints of the framework.

#### 3.2.2 Structural Analysis

To observe the surface morphology and particle shape, scanning electron microscopy (SEM) was employed. As shown in Figure 1A, both ZIF-8 and LAP@ZIF-8 exhibited well-defined hexagonal structures typical of ZIF-8.^40^ These images support successful nanoparticle formation with a uniform morphology. The apparent size of ZIF-8 was found to be 155.7 ± 54 nm, while that of LAP@ZIF-8 was 132.3 ± 30.7 nm, and the diameter of 100 nanoparticles was measured using the ImageJ program for this average particle size analysis based on SEM images. The size values calculated for ZIF-8 as a result of SEM were found to be comparable to similar results in the literature.^41^ In fact, the particle size and dispersion behavior of ZIF-8 and LAP@ZIF-8 nanoparticles were primarily evaluated using dynamic light scattering (DLS), which provides information on the hydrodynamic diameter and colloidal stability in aqueous environments. To evaluate batch-to-batch reproducibility and formulation quality, we performed dynamic light scattering (DLS) analysis on three independently synthesized batches of both ZIF-8 and LAP@ZIF-8 nanoparticles. The results showed minimal variation across batches, confirming the consistency of the one-pot synthesis approach. The average hydrodynamic diameter of ZIF-8 was 263.5 ± 4.8 nm, while LAP@ZIF-8 showed a size of 236.1 ± 2.4 nm. The polydispersity index (PDI) remained below 0.2 for both formulations (0.230 ± 0.016 for ZIF-8 and 0.173 ± 0.015 for LAP@ZIF-8), indicating good nanoparticle size uniformity and colloidal stability (Figure 1B). These results suggest that LAP loading slightly reduced particle size and enhanced monodispersity.^42^

**Figure 1:**
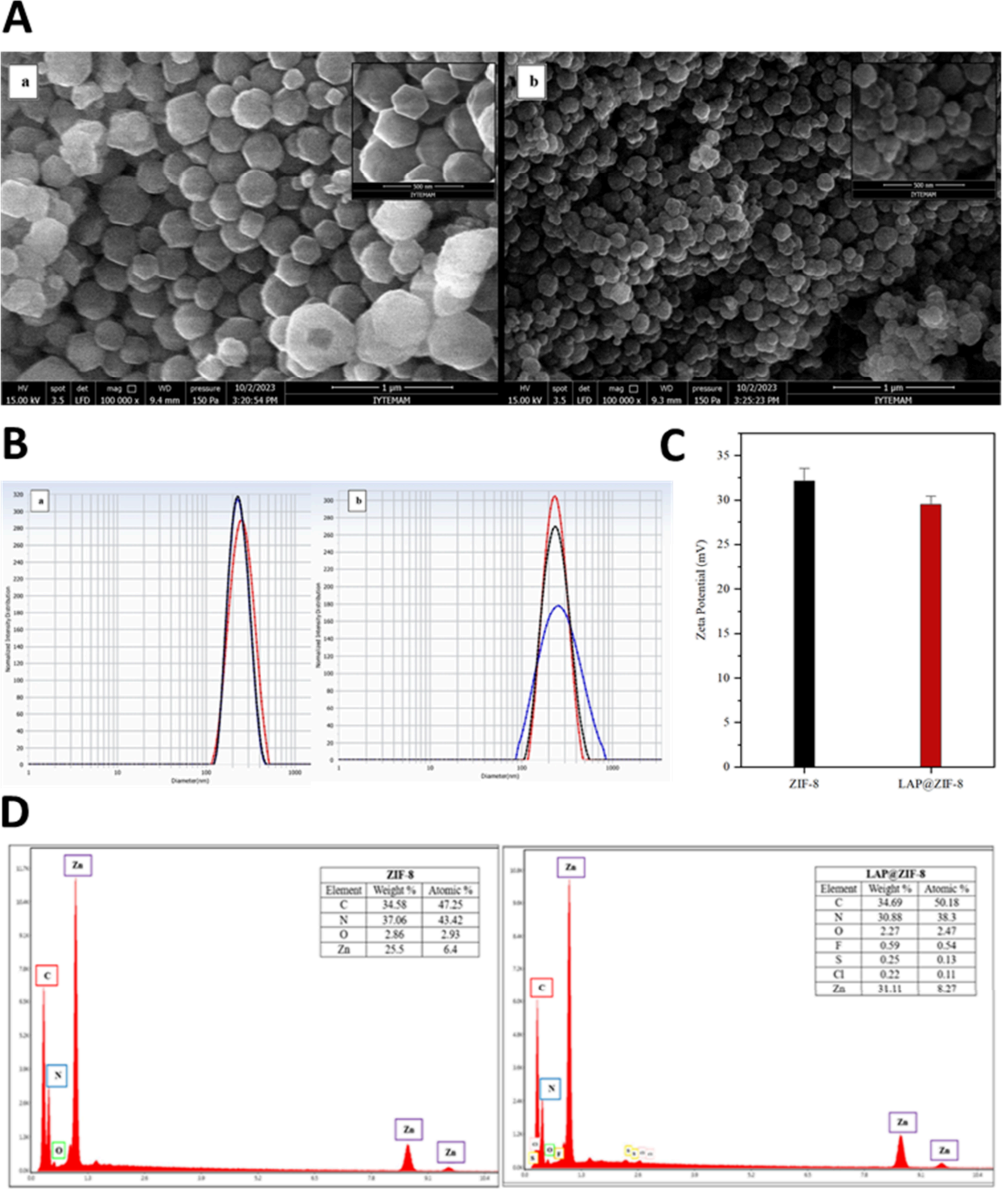
(A) SEM images of (a) ZIF-8 and (b) LAP@ZIF-8, (B) DLS measurements of (a) ZIF-8 and (b) LAP@ZIF-8, (C) zeta potentials of ZIF-8 and LAP@ZIF-8, and (D) EDX analysis of ZIF-8 and LAP@ZIF-8.

This observed size reduction may be explained by the influence of LAP during the crystal nucleation process.^40^ The presence of LAP molecules during synthesis likely facilitated faster nucleation rates and inhibited excessive crystal growth, leading to smaller, more uniform particles. This phenomenon is supported by previous findings where guest molecules modulate metal–ligand interactions during MOF formation. LAP’s functional groups may weakly interact with Zn^2+^ or 2-MeIm, subtly affecting the crystallization pathway. In another study, the hydrodynamic dimensions after LAP loading were lower than those of empty nanoparticles; this demonstrated that encapsulating a hydrophobic drug into the internal hydrophobic cavity of the nanostructure resulted in smaller particles.^43^ In addition, it is thought that the positively charged ZIF-8 surface may have been attracted to the negatively charged groups of LAP (−8.3 mV) and that clustering may have occurred between the two, causing a decrease in diameter. It has been reported in various articles that particle sizes below 200 nm are suitable for cancer cell uptake, while nanoparticles between 100 and 200 nm accumulate in cancerous tissues due to the EPR effect.^44,45^ This shows that the particle size of LAP@ZIF-8 is compatible with the literature.

Additionally, zeta potential measurements conducted across triplicate syntheses confirmed positive surface charges for both systems with values of +32.13 ± 1.18 mV for ZIF-8 and +29.49 ± 0.75 mV for LAP@ZIF-8, indicating good electrostatic stability in suspension (Figure 1C). These measurements, along with consistent particle size and PDI values, demonstrate excellent batch-to-batch reproducibility. The positive surface charge of ZIF-8 is attributed to the metal components (Zn^2+^) on its outer surface.^46^ An insignificant difference between zeta potential values was observed; it shows that LAP molecules coordinate with Zn^2+^ and are located within the nanoparticle. It is thought that the reason for the decrease in zeta potential is due to the negatively charged elements in the structure of LAP. This decrease also confirms the presence of LAP in the nanoparticle. It has been shown in the literature that the zeta potential values of nanoparticles obtained by encapsulating the chemical into ZIF-8 are similar to the value decrease after encapsulation^47^ and that ZIF-8 synthesized in another article also has similar values.^41,46^ In addition, studies in the literature have observed that the zeta value obtained as a result of nanoparticle encapsulation of LAP is a positive value.^39,41^ The cancer cell surface becomes negatively charged in the case of cancer, when negatively charged components such as phosphatidylserine, anionic phospholipids, glycoproteins, and proteoglycans in the inner layer of the cell membrane settle on the cell surfaces. Nanoparticles show a high affinity for the cell membrane, mainly due to electrostatic interactions. After the adsorption of nanoparticles to the cell membrane, uptake occurs through several possible mechanisms, such as pinocytosis, nonspecific or receptor-mediated endocytosis, or phagocytosis. Positively charged nanoparticles are preferentially taken up by tumors. Studies show that positively charged nanoparticles bind to the negatively charged surface of tumor endothelial cells through electrostatic interactions. A higher cellular uptake was shown, where electrostatic interactions between the negatively charged membrane and positively charged nanoparticles facilitate uptake.^48^

The EDX analysis of ZIF-8 and LAP@ZIF-8 is shown in Figure 1D. While characteristic elements such as carbon (C), oxygen (O), nitrogen (N), and zinc (Zn) were seen in the EDX spectrum of ZIF-8, the presence of fluorine (F), sulfur (S), and chlorine (Cl) elements found in LAP was also seen in LAP@ZIF-8. The EDX spectra of the LAP@ZIF-8 confirmed the incorporation of LAP into ZIF-8, as shown by the detection of F, S, and Cl elements. The existence of all used elements in the nanoparticles and the absence of any pending impurities were confirmed by EDX analysis. These results showed the absence of contaminants with confirmation of the elemental composition of LAP in formulated LAP@ZIF-8.

The measurement of FTIR was performed for LAP, ZIF-8, and LAP@ZIF-8. The FTIR spectrum of the samples on the 400–4000 cm^–1^ absorption band is shown in Figure 2A. The FTIR spectrum of pure lapatinib exhibits characteristic bands at ∼3150–3050 cm^–1^ (N–H), 3017 cm^–1^ (aromatic C–H), 1690 cm^–1^ (C=N), 1312 cm^–1^ (S=O), 1264 cm^–1^ (C–O), 1160 cm^–1^ (furan C–O–C), 1250–1120 cm^–1^ (C–SO_2_), 1031 cm^–1^ (C–F), 679 cm^–1^ (C=C bend), and 564 cm^–1^ (C–Cl), consistent with a literature report.^49^ It presented remarkable bands at 3136, 2932, 1635, 1584, 1456, 1425, 1383, 1311, 1146, 995, 759, 694, and 420 cm^–1^ for the ZIF-8 sample. The peaks were observed at 3136, 2932, 1635, and 1584 cm^–1^ corresponding to aromatic C–H asymmetric stretching, aliphatic C–H asymmetric stretching, C=C stretching, and C=N stretching vibrations of imidazole, respectively. The bands in the spectral region of 1460–1300 cm^–1^ (1456, 1425, 1383, and 1311 cm^–1^) are associated to the ring stretching, whereas the band at 1146 cm^–1^ is associated to aromatic C–N stretching mode. The peak at 995 cm^–1^ could be assigned to a C–N bending (in-plane ring bending) vibration. The peaks at 759 and 694 cm^–1^ were associated with the C–H bending mode and ring out-of-plane bending vibration of the 2-MeIm, respectively.

**Figure 2:**
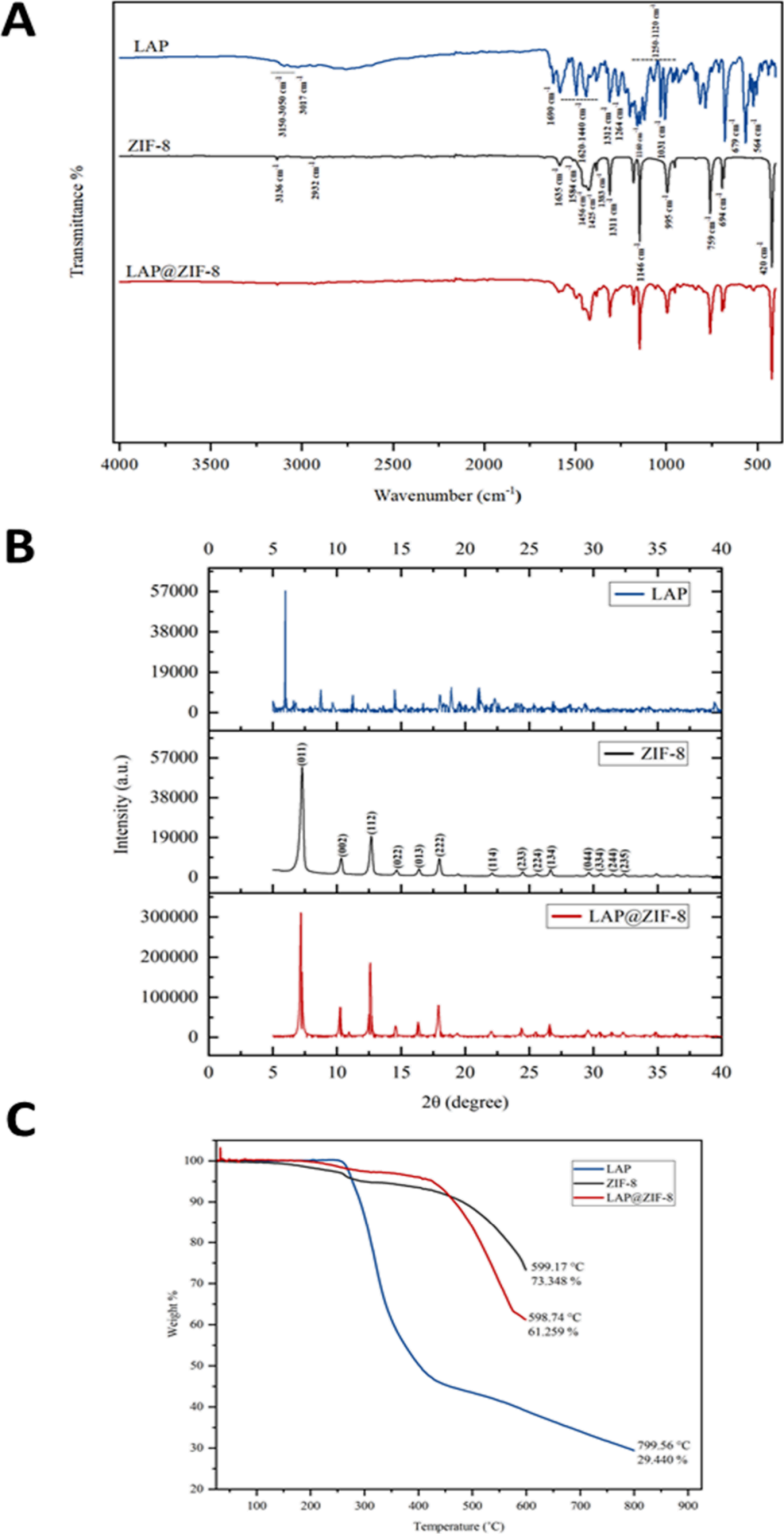
(A) FTIR spectra of LAP, ZIF-8, and LAP@ZIF-8; (B) XRD patterns of LAP, ZIF-8, and LAP@ZIF-8; (C) TGA curves of LAP, ZIF-8, and LAP@ZIF-8.

Combination of Zn and N to form the imidazolate was confirmed by observing the Zn–N stretching vibration band at 420 cm^–1^. The results match the study in the literature and demonstrate successful synthesis of ZIF-8.^50^ Several peaks such as 564 cm^–1^ of LAP disappeared in the spectra of LAP@ZIF-8, indicating that the peaks were covered by other components. Several peaks in the nanoparticle were shifted in the spectrum, suggesting that there was interaction among components in the formation of LAP@ZIF-8. When the LAP@ZIF-8 spectrum is examined carefully, it can be said that the intensity and sharpness of the peaks have increased. When the spectra obtained for ZIF-8 were compared with LAP@ZIF-8, it was noticed that the spectra of the two samples were very similar. Considering the FTIR spectra, LAP@ZIF-8 overlaps with the spectra of ZIF-8 but does not completely overlap with the spectra of LAP. This result suggests the successful incorporation of LAP into ZIF-8 molecules. Due to the encapsulation within the ZIF-8 framework, the characteristic peaks of LAP are masked, and this encapsulation also protects the drug from degradation caused by the environment.^51^ These results confirm that the synthesized nanoparticle contains both ZIF-8 and LAP and LAP was successfully loaded in ZIF-8. The FTIR spectra of LAP@ZIF-8 showed notable changes compared to pure ZIF-8 and LAP, such as shifts in absorption bands corresponding to −NH, −C=N, and −SO_2_ groups. These subtle spectral shifts suggest the presence of weak chemical interactions, likely involving hydrogen bonding or π–π stacking between LAP and the framework’s organic linkers. However, no new peaks were observed, indicating that no covalent bond formation occurred. Rather, the results support physical encapsulation stabilized by noncovalent interactions. This interpretation is further supported by the XRD pattern of LAP@ZIF-8, where the characteristic crystalline peaks of ZIF-8 were retained, albeit with reduced intensity. The absence of major peak shifts confirms that the overall framework structure was preserved, while the decreased crystallinity suggests a partial interaction or confinement of LAP within the porous structure.

The crystal structures of LAP, synthesized ZIF-8, and LAP@ZIF-8 were identified by XRD, and the results are shown in Figure 2B. Peak positions and diffraction peaks of their crystals are shown between 2θ values between 5 and 40°. A very sharp peak at 7.28° was observed in the XRD pattern of the ZIF-8, indicating that a highly crystalline material was achieved. The characteristic diffraction peaks at 2θ = 7.28, 10.34, 12.68, 14.67, 16.40, 18.00, 22.12, 24.48, 26.67, and 29.66° for the ZIF-8 sample were observed, which can be assigned to the (011), (002), (112), (022), (013), (222), (114), (233), (134), and (044) planes, respectively.^50^ ZIF-8 nanoparticles showed strong peaks, which are in good agreement with previously reported findings.^15–17^ The other weak peaks at 2θ = 25.58, 30.59, 31.52, and 32.38° for the ZIF-8 sample were observed, which can be assigned to the (224), (334), (244), and (235) planes, respectively.^52^ The XRD result of LAP showed much more frequent sharp peaks at higher intensity, while that of LAP@ZIF-8 showed sharp but less intense peaks. It was observed that after LAP loading, the characteristic diffraction peaks of ZIF-8 were weakened, but the main characteristic peaks were essentially unchanged. XRD analysis revealed the crystal structure of LAP@ZIF-8 and showed that the addition of LAP did not significantly change the ZIF-8 values. The preservation of peak positions indicates that the crystal structure of ZIF-8 remains stable after LAP loading, suggesting that the drug was physically encapsulated or surface-adsorbed without altering the framework’s lattice parameters. Interestingly, the LAP@ZIF-8 pattern exhibited higher peak intensities than ZIF-8 alone, which may be attributed to improved crystallinity, enhanced particle ordering, or slight variations in sample preparation and packing density. The absence of distinct LAP peaks in the LAP@ZIF-8 pattern is likely due to the low drug loading content (6.55%) and the amorphous or molecularly dispersed state of LAP within the MOF structure. These observations are consistent with previous studies reporting that drug molecules embedded in porous hosts may not produce detectable diffraction signals if they are present in noncrystalline or low-concentration forms.

The TGA nalysis was performed between 25 and 800 °C for LAP and 25–800 °C for ZIF-8 and LAP@ZIF-8 at a constant heating rate of 10 °C/min under an air atmosphere. Figure 2C exhibits the TGA curves of LAP, ZIF-8, and LAP@ZIF-8. The initial weight loss is attributed to evaporation of adsorbed moisture, while subsequent losses are associated with decomposition of organic components, in agreement with previous reports on nanoparticle and MOF systems.^15,33^ TGA analysis showed that LAP, ZIF-8, and LAP@ZIF-8 samples were stable below ∼100 °C. It was observed that the thermal decomposition of LAP started at 245 °C, and the maximum weight loss was 70.56% at 799.56 °C, while the thermal decomposition of ZIF-8 started at 250 °C, and the maximum weight loss was 26.652% at 599.17 °C. It was observed that the thermal decomposition of LAP@ZIF-8 started at 420 °C and the maximum weight loss of 38.741% occurred. The TGA curve for the thermal degradation process of LAP@ZIF-8 has three stages. The LAP@ZIF-8 was stable up to 420 °C and had very minor changes in weight loss, which is 4.625%. In the second stage of decomposition, starting at 420 to 560 °C, this weight loss is 27.814%. In the third stage of decomposition, starting at 560–598.74 °C, this weight loss is 6.265%. The situation after the loss of water molecules of ZIF-8 indicates the loss of LAP molecules because there is no weight loss of ZIF-8 in this temperature range. The weight loss can be attributed to the decomposition of LAP that is encapsulated in the ZIF-8 frameworks. The latter degradation state is due to thermal degradation of LAP and ZIF-8 along with their carbonization. This weight loss may have been caused by the release of water molecules and other absorbed unreacted molecules, such as 2-MeIm, from the pore structure. Subsequently, with the increase in temperature, the skeleton structure of the sample collapses and decomposes, and the structural integrity of the crystal is destroyed. ZIF-8 and LAP@ZIF-8 samples decompose, and zinc oxide is formed. The decreased weight loss of LAP suggests that LAP interacts with ZIF-8 through electrostatic interactions and coordination reactions. Encapsulation of LAP in ZIF-8 is clearly evident from the thermal curve of LAP@ZIF-8. All of these results illustrate that the LAP@ZIF-8 sample has good thermal stability.

#### 3.2.3 Drug Release Study

The ability of the nanocarrier to efficiently release the drug at the desired site is an important feature of the delivery system. Figure 3 represents the release of LAP from LAP@ZIF-8 in pH 5.5 and pH 7.4. LAP@ZIF-8 showed a controlled release profile in the release environment. At the end of 96 h, 76.99 and 42.75% of LAP were released in pH 5.5 and pH 7.4, respectively. The observed pH-dependent release behavior is attributed to the pH sensitivity of the ZIF-8 framework, which is composed of zinc ions (Zn^2+^) coordinated to 2-methylimidazole linkers. Under physiological or neutral conditions (pH 7.4), ZIF-8 remains structurally stable due to strong metal–ligand coordination. However, in acidic environments (such as endosomal pH ∼5.5), protonation of the imidazolate ligands weakens the Zn–N bonds, triggering gradual degradation of the framework. This facilitates the selective intracellular release of LAP in cancer cells following endocytosis. Thus, the enhanced drug release at pH 5.5 reflects the acid-triggered disassembly of LAP@ZIF-8, enabling controlled and site-specific delivery while minimizing premature release in circulation. This pH-triggered release mechanism has been widely reported in the literature for ZIF-8-based nanocarriers, which exhibit accelerated drug release under mildly acidic conditions,^53^ consistent with tumor or intracellular environments.

**Figure 3:**
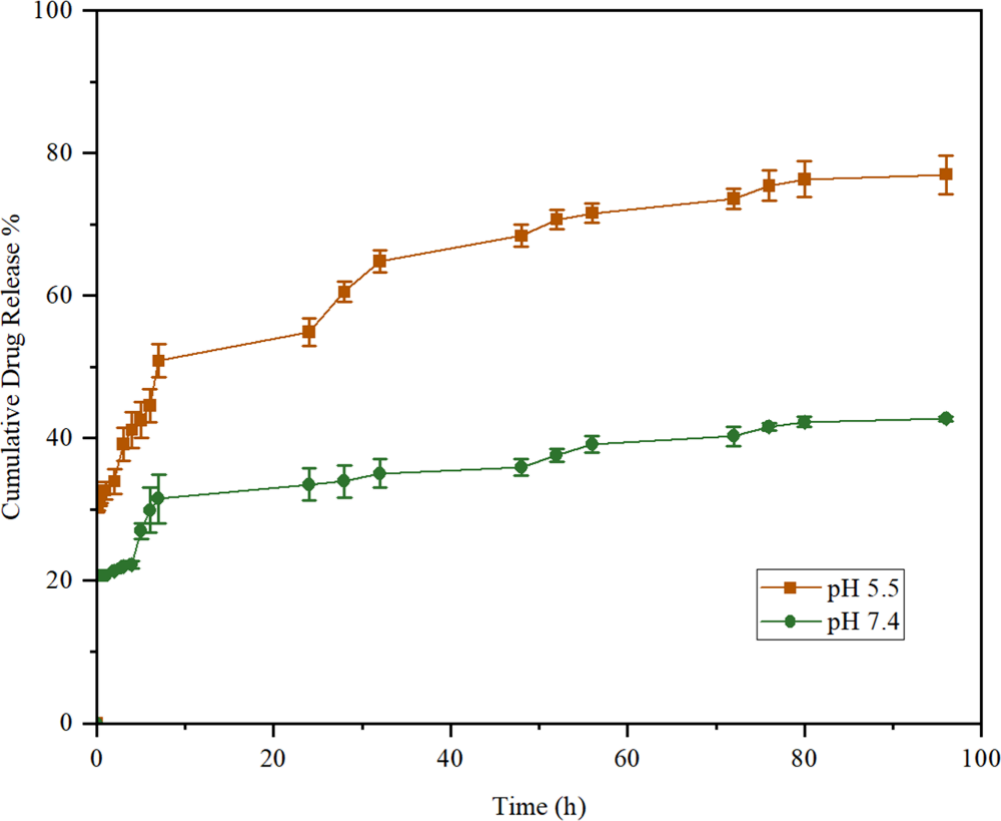
Cumulative drug release profile of LAP@ZIF-8 in media of different pH values for 96 h.

The monitoring period of 96 h was selected based on previous studies indicating that ZIF-8 frameworks typically degrade under acidic conditions within 3–5 days, during which the majority of encapsulated drug is released. This window is sufficient to capture both the initial burst and sustained release phases of LAP. For instance, Wang et al. reported that over 80% of encapsulated celastrol was released from ZIF-8 within 96 h at pH 5.5, highlighting this period as an appropriate benchmark for assessing pH-responsive release behavior of ZIF-based nanocarriers.^42^ Therefore, our choice of a 96 h evaluation period is supported by an established precedent in the literature.

To further validate our *in vitro* release setup and address potential artifacts introduced by mechanical agitation, we conducted a control study comparing release profiles under stirring (100 rpm) and static conditions at both pH 5.5 and 7.4 (data are not shown). Drug release was monitored over a 72 h period. As expected, slightly reduced release rates were observed in the absence of stirring, especially during the early phase, likely due to diminished diffusion and less effective sink conditions. Nevertheless, the overall release trends remained consistent between the stirred and static setups. These findings support the use of gentle agitation to maintain assay reliability and reproducibility without significantly altering the fundamental release behavior of LAP@ZIF-8.

LAP was released continuously for up to 96 h, indicating that LAP was encapsulated in the hydrophobic core of the nanoparticle, leaving almost nothing on the surface. The slower release at pH 7.4 compared to that at pH 5.5 is beneficial for cancer cell targeting and higher tumor cell inhibition. The slow and relatively low release of LAP at the body’s physiological pH of pH 7.4 also helps reduce its toxicity on normal tissues. Based on the findings, it is hypothesized that LAP release can be controlled at pH 7.4 and remains stable in ZIF-8. Additionally, the solubility of LAP increases at pH 5.5 due to increased protonation of the amino groups in LAP molecules.^54,55^ This slow release in pH 7.4 indicated that the hydrophobic pores of ZIF-8 had assisted slow release of hydrophobic LAP. The fast release in pH 5.5 was due to the disintegration of the ZIF-8 structure in acidic pH. While pH 5.5 is commonly used to mimic the acidic environment of endosomal or lysosomal compartments following cellular uptake, it is important to note that the extracellular tumor microenvironment generally exhibits a milder acidity, typically in the range of pH 6.5–6.8. Therefore, the current results may overestimate the rate of drug release under *in vivo* tumor conditions. In future work, the release profile of LAP@ZIF-8 should be further evaluated at intermediate pH levels (e.g., pH 6.5) to more accurately model the conditions found in the tumor interstitium. This would help determine whether the platform maintains pH sensitivity in environments relevant to extracellular tumor targeting, in addition to intracellular release mechanisms. In general, sustained release exposes cancer cells to the drug continuously, providing an increased likelihood of cell death. This can also reduce drug dose and dosing frequency and increase therapeutic effectiveness in cancer treatment.^56^ In conclusion, the release of LAP from LAP@ZIF-8 after internalization may result in enhanced cytotoxic activity against cancer cells.

#### 3.2.4 Stability of LAP@ZIF-8 Nanoparticles

To assess the long-term physicochemical stability of the LAP@ZIF-8 formulation, we monitored the particle size, PDI, zeta potential, and free drug leakage over 30 days of storage at 4 °C. As shown in Table 1, LAP@ZIF-8 nanoparticles exhibited excellent stability throughout the study period. The hydrodynamic diameter remained relatively consistent, increasing only slightly from 236.0 ± 2.1 nm on day 1 to 243.3 ± 2.7 nm on day 30. Similarly, PDI values remained below 0.25 across all time points (e.g., 0.19 ± 0.01 on day 1 and 0.23 ± 0.01 on day 30), indicating good colloidal uniformity and minimal aggregation. Zeta potential measurements showed a gradual decrease in surface charge from +29.1 ± 1.3 to +26.2 ± 1.3 mV, consistent with typical surface relaxation or minor ionic exchange under storage conditions. Importantly, cumulative free drug release remained low, with only 6.7 ± 0.6% of LAP detected in the supernatant by day 30. These findings confirm that LAP@ZIF-8 maintains its structural integrity, drug loading capacity, and colloidal stability under refrigerated storage for at least one month. This performance supports the potential of LAP@ZIF-8 as a robust and storage-stable nanocarrier for further preclinical development.

**Table 1:** Physicochemical Stability of LAP@ZIF-8 Nanoparticles during 30 Days of Storage at 4 °C^a^

day	free LAP (%)	size (nm)	PDI	zeta potential (mV)
1	1.9 ± 0.3	236 ± 2.1	0.17 ± 0.01	29.1 ± 1.3
3	2.1 ± 0.2	237.4 ± 2.3	0.19 ± 0.02	28.6 ± 1.2
5	2.6 ± 0.3	238.9 ± 2.4	0.2 ± 0.02	28 ± 1.0
7	3.1 ± 0.4	239.5 ± 2.3	0.21 ± 0.01	27.6 ± 1.1
15	4.8 ± 0.5	241.1 ± 2.7	0.22 ± 0.02	26.8 ± 1.2
30	6.7 ± 0.5	243.3 ± 2.6	0.23 ± 0.01	26.2 ± 1.3

a Values are expressed as the mean ± standard deviation (*n* = 3).

#### 3.2.5 Biocompatibility Assay: Serum Protein Binding

Serum albumin is the most abundant in blood, and human serum albumin (HSA) and BSA are the most studied proteins. The interaction of drugs with serum albumin may affect their pharmacokinetic and pharmacodynamic properties, distribution in the body, passage through biological membranes, severity of pharmacological effects, and elimination rates. Drugs can be present in the circulatory system either bound to plasma protein or in a free/unbound state. The drug bound to the plasma protein is not pharmacologically active. Drugs with strong binding affinity to serum albumin may cause undesirable effects, such as causing a longer half-life of a drug in the body, thus reducing its value as a therapeutic. The unbound drugs interact with their therapeutic targets and exert their effects.^57^

In the serum–protein binding study, the samples were centrifuged with FBS and the binding percentages to serum proteins were calculated based on the unbound protein remaining in the supernatant. Since the amount of plasma protein may vary from person to person, experiments were carried out using different serum and sample concentrations. Table 2 shows the protein binding percentages. It appears that there is no linear increase or decrease according to serum/sample ratios and the increase in serum ratio does not have a significant effect on protein binding. When the study by Semete et al.^35^ was examined, serum–protein binding to nanoparticles was around 40%, and in this study, the highest binding was found to be about 10.39% in LAP@ZIF-8. Based on the literature information and the obtained trial data, it is expected that the drug will have good pharmacokinetic distribution and accumulate concentratedly in the targeted tissues. It is thought that the nanoparticle can be delivered to the target tissue at high rates due to its nonprotein binding or low binding results. When the results are evaluated, it is thought that the samples, especially the LAP@ZIF-8, are biocompatible since the protein binding percentages are low.

**Table 2:** Protein Binding Percentages of LAP, ZIF-8, and LAP@ZIF-8^a^

sample	*V*_FBS_:*V*_Sample_	protein binding (%)
LAP	10:90	n.d.
	20:80	n.d.
	30:70	n.d.
	40:60	n.d.
	50:50	5.36 ± 0.57
	60:40	9.45 ± 2.80
	70:30	15.99 ± 0.44
	80:20	12.26 ± 1.94
	90:10	11.53 ± 0.62
ZIF-8	10:90	n.d.
	20:80	n.d.
	30:70	n.d.
	40:60	n.d.
	50:50	10.46 ± 7.01
	60:40	19.26 ± 3.44
	70:30	15.55 ± 5.54
	80:20	14.11 ± 5.89
	90:10	14.48 ± 8.45
LAP@ZIF-8	10:90	n.d.
	20:80	n.d.
	30:70	n.d.
	40:60	n.d.
	50:50	6.50 ± 2.10
	60:40	6.87 ± 1.65
	70:30	10.39 ± 1.47
	80:20	8.32 ± 2.79
	90:10	5.57 ± 0.90

a n.d., Not determined.

#### 3.2.6 Biocompatibility Assay: Hemocompatibility (Hemolysis)

Hemolysis is the breakdown (lysis) of red blood cells and the release of their contents (cytoplasm) into the surrounding fluid. Nanoparticles can easily reach the circulation due to their size and route of administration, and red blood cells (erythrocytes) may be the first biological entity with which they come into contact with. The hemolysis assay is used to evaluate nanoparticle toxicity resulting from the interaction of nanoparticles with red blood cells. This assay aims to determine the interaction of nanoparticles with the red blood cell membrane and the percentage of released hemoglobin (Hb).^58^ Evaluation of the ability of nanoparticles to integrate with blood is described as nanoparticle compatibility. Hemolytic activity (% hemolysis) is calculated by dividing the released hemoglobin concentration by the total hemoglobin concentration in exposed red blood cells. Accordingly, 0–2% hemolysis is nonhemolytic, 2–5% is slightly hemolytic, and more than 5% is hemolytic.^58^ An *in vitro* hemocompatibility study was performed. The percentages of hemolysis results in the experimental groups remained below 2%, which is lower than the 5% acceptable hemolysis limit reported for biomaterials in contact with blood (Figure 4). This research showed that the samples showed that in the case of blood contact, blood hemolysis did not exceed 5% of the positive control and there was no hemolytic effect. The hemolytic values of LAP@ZIF-8 were determined as 1.08 ± 0.13, 1.44 ± 0.21, and 1.68 ± 0.18% according to the increasing concentration value, indicating that the hemolytic toxicity of LAP was reduced when encapsulated into ZIF-8. The experimental result showed that LAP@ZIF-8 is hemocompatible, harmless to fresh blood (does not show any damage to the red blood cell membrane), and can be used in practical applications related to biological aspects.

**Figure 4:**
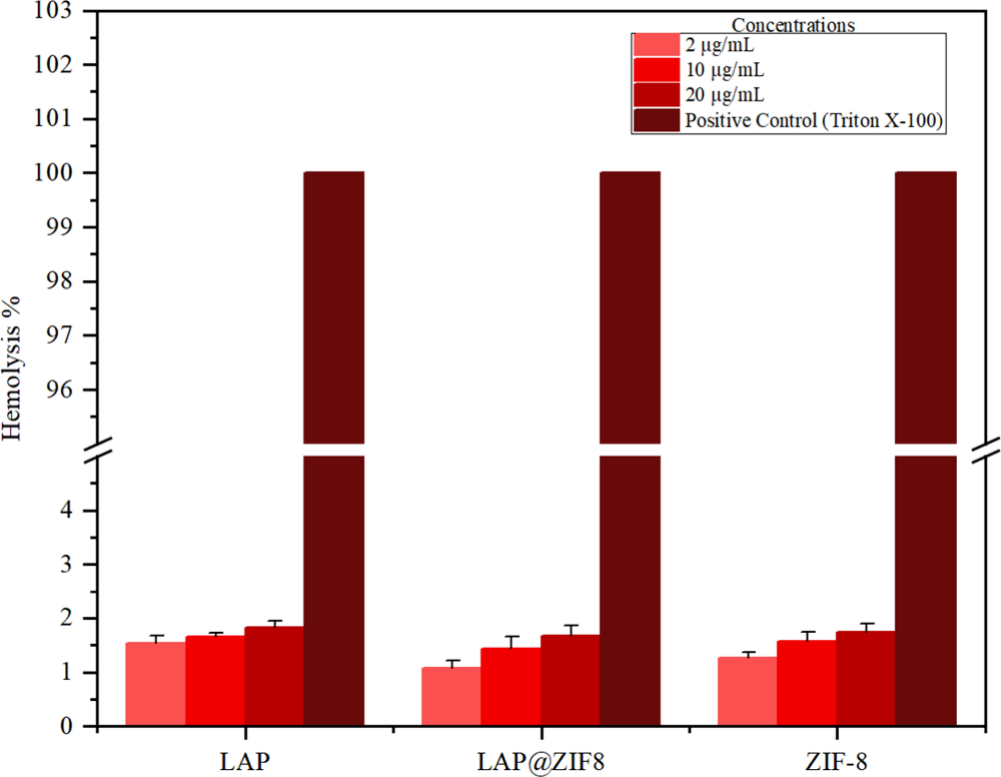
Hemolysis percentages of LAP, ZIF-8, and LAP@ZIF-8.

### 3.3 *In Vitro* Investigation of Cancer Activity

The cytotoxicities of LAP, ZIF-8, and LAP@ZIF-8 against SKBR-3 and MCF-7 cancer cell lines and also the MCF-10A noncancer cell line, and consequently the potential of ZIF-8 used as a drug carrier, were evaluated using the MTT assay (Figures 5, 6, and 7). It appears that the blank nanocarrier, ZIF-8, does not produce any cytotoxic effects on cancer cells. As shown in Figures 5d and 6d, ZIF-8 exhibited a limited effect on the proliferation of both cell lines. Although there was no significant difference, it was observed to be more lethal than SKBR-3 in MCF-7 cells. The fact that cell viability remained above 60% even at the highest concentration tested (100 μg/mL) for two cell lines in addition to almost no cell death at concentrations below this concentration indicates that ZIF-8 nanoparticles have low cytotoxicity and good biocompatibility. It has been shown in the literature that ZIF-8 has no significant cytotoxicity up to 30 μg/mL, and cytotoxicity above 30 μg/mL is due to the effect of released Zn^2+^ on mitochondrial ROS production.^59^

**Figure 5:**
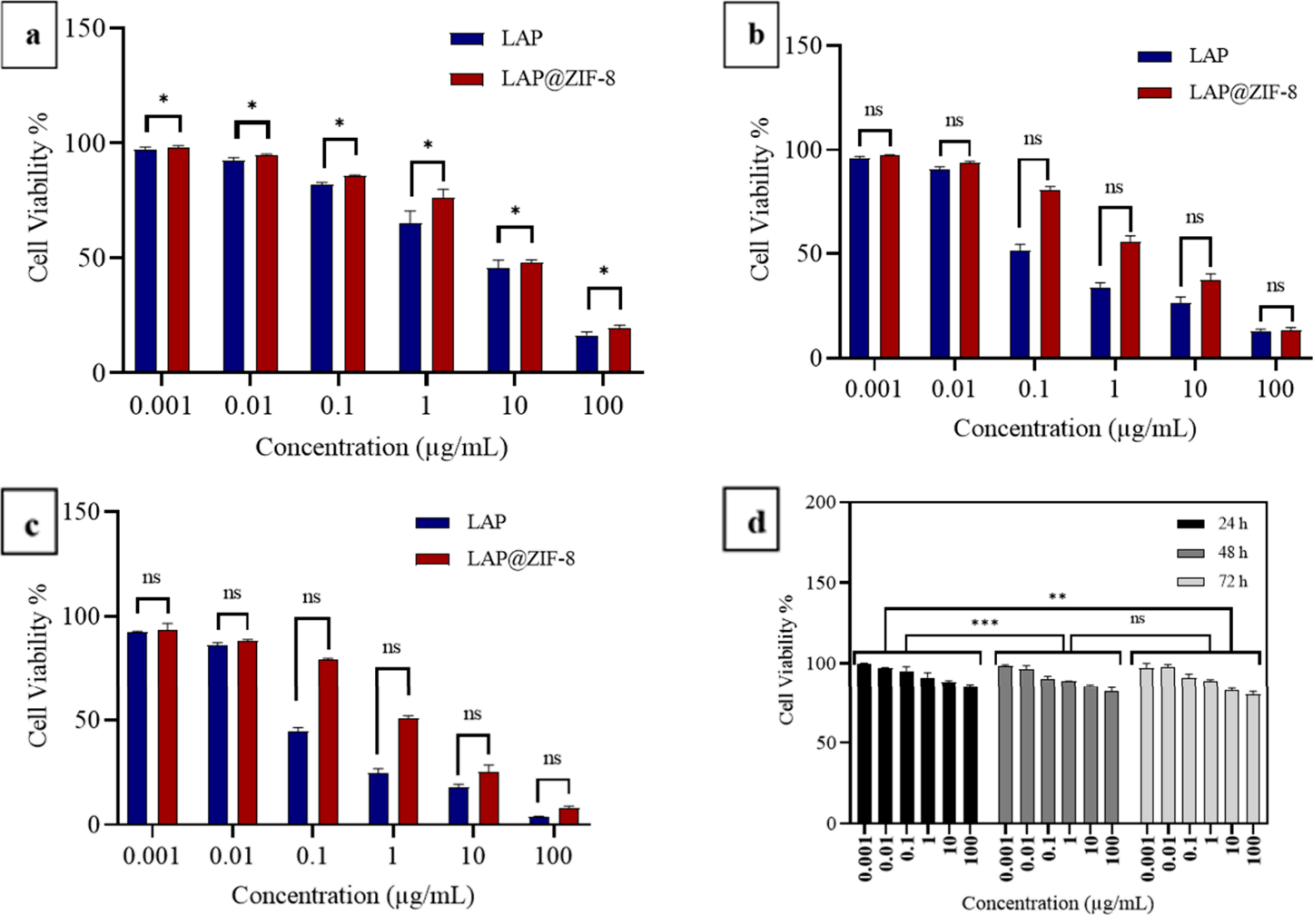
*In vitro* cytotoxicity profiles of LAP, ZIF-8, and LAP@ZIF-8 against the SKBR-3 cell line at various concentrations. *In vitro* cytotoxicity profile of LAP and LAP@ZIF-8 after (a) 24 h, (b) 48 h, and (c) 72 h of incubation time as assayed by MTT. *In vitro* cytotoxicity profile of (d) ZIF-8. Significantly different data were indicated by asterisks (*p* < 0.05 (*), *p* < 0.01 (**), *p* < 0.001 (***), and *p* < 0.0001 (****); ns indicates nonsignificant).

**Figure 6:**
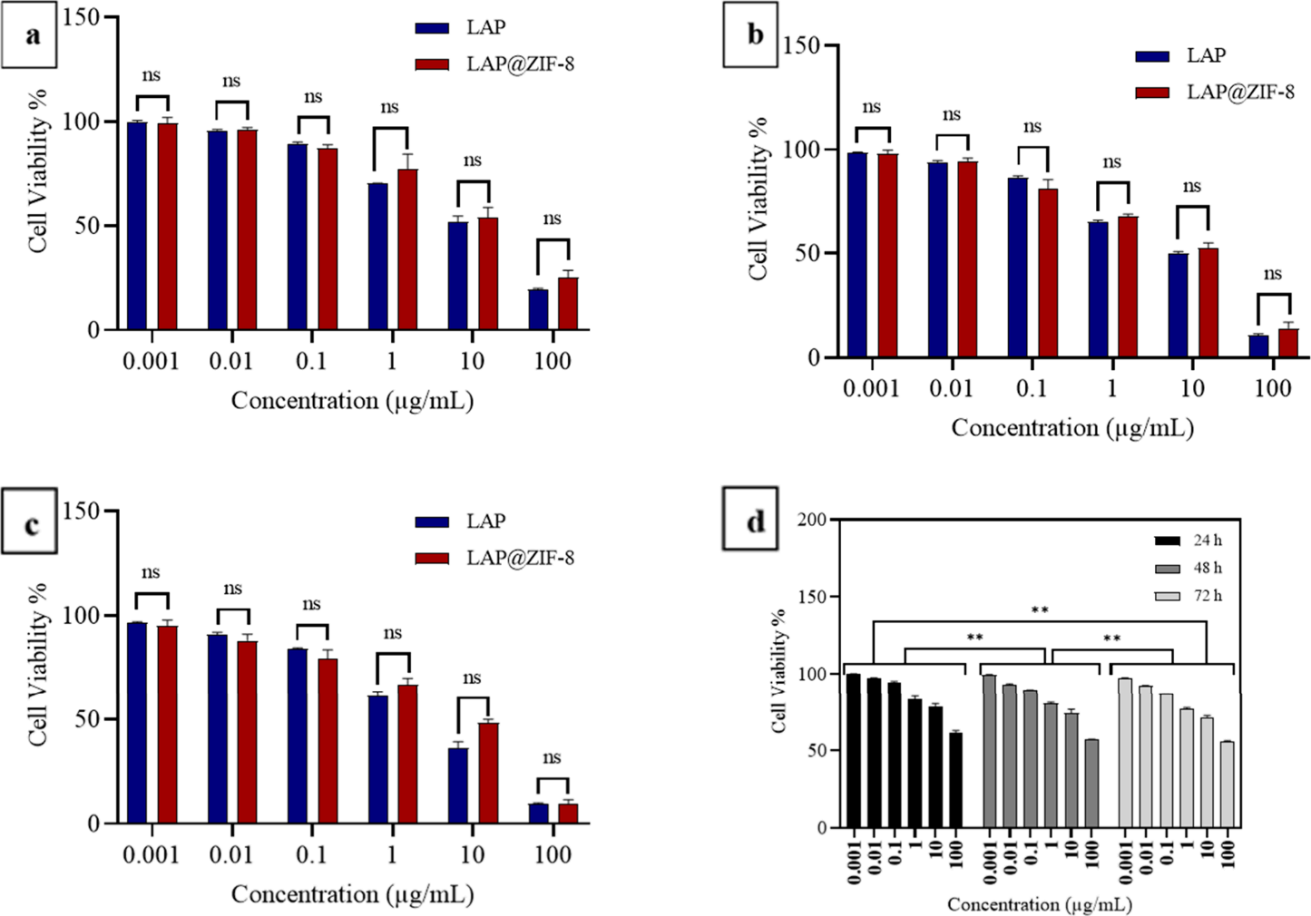
*In vitro* cytotoxicity profile of LAP, ZIF-8, and LAP@ZIF-8 against the MCF-7 cell line at various concentrations. *In vitro* cytotoxicity profiles of LAP and LAP@ZIF-8 after (a) 24 h, (b) 48 h, and (c) 72 h of incubation time as assayed by MTT. *In vitro* cytotoxicity profile of (d) ZIF-8. Significantly different data were indicated by asterisks (*p* < 0.05 (*), *p* < 0.01 (**), *p* < 0.001 (***), and *p* < 0.0001 (****); ns indicates nonsignificant).

**Figure 7:**
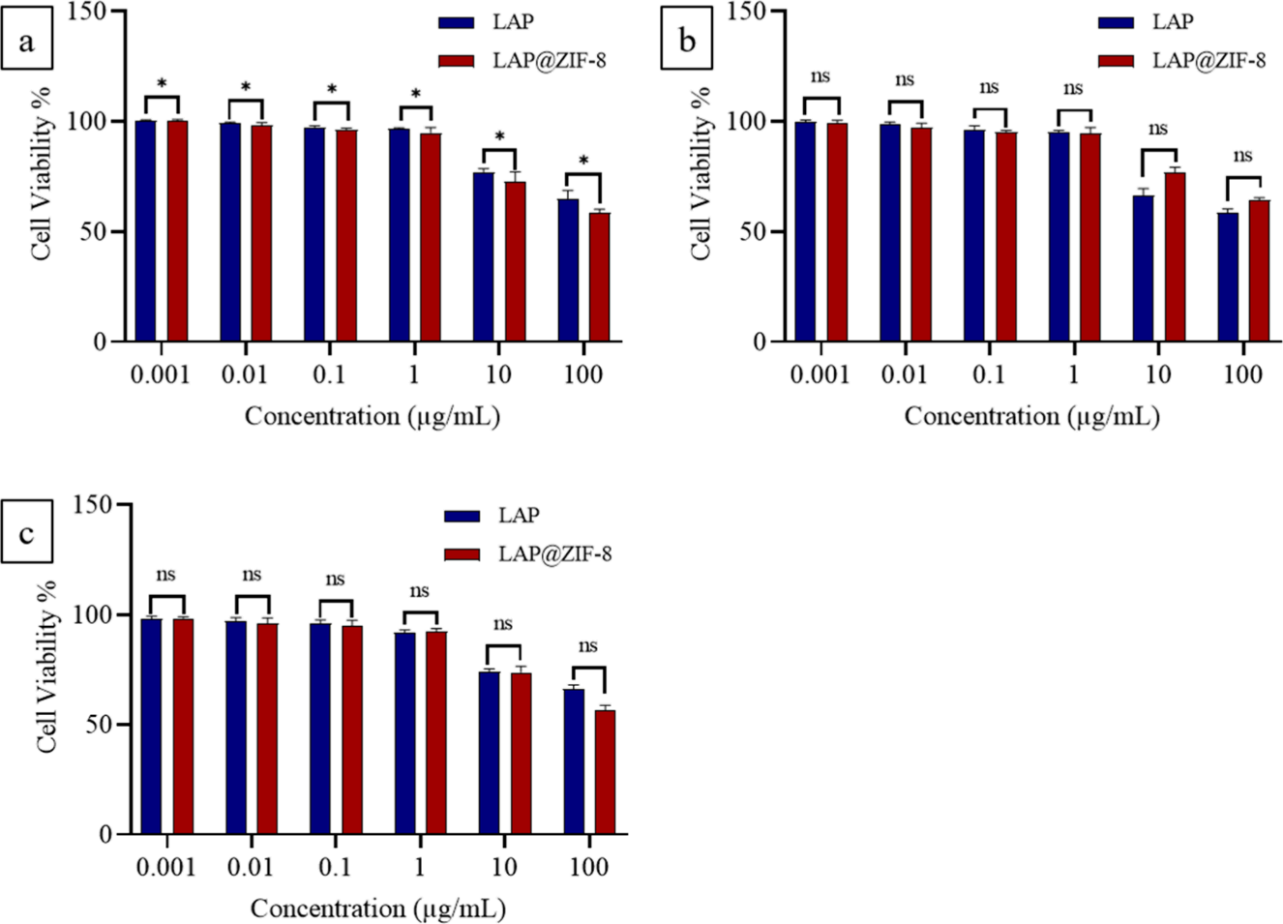
*In vitro* cytotoxicity profile of LAP, ZIF-8, and LAP@ZIF-8 against the MCF-10A cell line at various concentrations. *In vitro* cytotoxicity profile of LAP and LAP@ZIF-8 after (a) 24 h, (b) 48 h and (c) 72 h incubation times as assayed by MTT. Significantly different data were indicated by asterisks (*p* < 0.05 (*), *p* < 0.01 (**), *p* < 0.001 (***), and *p* < 0.0001 (****); ns indicates nonsignificant).

When the results were examined, it was seen that ZIF-8 maintained its viability and was compatible with the literature.^60^ The cytotoxic effect of free LAP and LAP@ZIF-8 was tested in SKBR-3 (Figure 5a–c) and MCF-7 (Figure 6a–c) cancer cells depending on the incubation time. As shown in figures, both formulations showed typical time- and concentration-dependent cytotoxicity in cancer cells. It was observed that there was no statistically significant difference between LAP and LAP@ZIF-8 treatment in MCF-7 cells within the investigated time periods. In fact, it is possible to say the same for the SKBR-3 cells. The IC_25_ (concentration of inhibitory sample that causes 25% inhibition) and IC_75_ (concentration of inhibitory sample that causes 75% inhibition) values (Table 3) and IC_50_ values (Table 4) were calculated and shown as a result of 24, 48, and 72 h of treatment of samples in SKBR-3 and MCF-7 cell lines.

**Table 3:** IC_25_ and IC_75_ Values of LAP, ZIF-8, and LAP@ZIF-8 for SKBR-3 and MCF-7 Cell Lines

	IC_25_ (μg/mL)						IC_75_ (μg/mL)					
	SKBR-3 cell line			MCF-7 cell line			SKBR-3 cell line			MCF-7 cell line		
incubation time	LAP	ZIF-8	LAP@ZIF-8	LAP	ZIF-8	LAP@ZIF-8	LAP	ZIF-8	LAP@ZIF-8	LAP	ZIF-8	LAP@ZIF-8
24 h	0.47	>100	1.50	0.78	26.95	1.94	73.53	>100	82.57	84.94	>100	>100
48 h	0.05	>100	0.31	0.59	10.36	0.52	18.22	>100	56.25	67.00	>100	74.52
72 h	0.03	>100	0.24	0.46	4.32	0.41	0.98	>100	11.38	47.15	>100	63.72

**Table 4:** IC_50_ Values of LAP, ZIF-8, and LAP@ZIF-8 for SKBR-3 and MCF-7 Cell Lines

	IC_50_ (μg/mL)					
	SKBR-3 cell line			MCF-7 cell line		
incubation time	LAP	ZIF-8	LAP@ZIF-8	LAP	ZIF-8	LAP@ZIF-8
24 h	7.96	>100	9.38	14.99	>100	22.05
48 h	0.19	>100	3.81	9.98	>100	16.13
72 h	0.09	>100	1.20	5.04	>100	9.14

According to IC_50_ values, LAP was much more toxic on SKBR-3 cells than on the MCF-7 cell line in the concentration range examined. In addition, LAP@ZIF-8 was significantly more toxic on SKBR-3 (HER2-positive) cells than on the MCF-7 (HER2-negative) cell line over the concentration range investigated. This demonstrated the effectiveness of LAP@ZIF in targeting SKBR-3 cells where HER2 was overexpressed. The IC_50_ values for compounds LAP, ZIF-8, and LAP@ZIF-8 are listed and indicate that in both SKBR-3 and MCF-7 cell lines, the IC_50_ value of ZIF-8 exceeded 100 μg/mL in all incubation times. After LAP was loaded onto ZIF-8 in the SKBR-3 cell line, the IC_50_ values of LAP@ZIF-8 were 9.38, 3.81, and 1.20 μg/mL after 24, 48, and 72 h incubation times, respectively. After LAP was loaded onto ZIF-8 in the MCF-7 cell line, the IC_50_ values of LAP@ZIF-8 were 22.05, 16.13, and 9.14 μg/mL after 24, 48, and 72 h incubation times, respectively. Overall, a comparison of IC_50_ values of the LAP@ZIF-8 compound with LAP revealed that the LAP@ZIF-8 showed an IC_50_ value close to the IC_50_ value of LAP in both SKBR-3 and MCF-7 cell lines. The lower cytotoxicity of LAP@ZIF-8 compared with LAP can be explained by the slow release of the drug. The situation encountered as a result of the experiment is supported by similar results in the literature and explained by similar reasons such as drug release, encapsulation, and loading. In one study, the cytotoxicities of DOXO (doxorubicin), ZIF-8, and the DOXO-ZIF-8 complex determined by the MTT assay were evaluated. It was observed that the DOXO-ZIF-8 complex had a higher IC_50_ value than free DOXO. It has been mentioned that the weaker cytotoxicity of DOXO-ZIF-8 compared to DOXO can be explained by the slow release of the drug.^61^ In another study, the cytotoxicities of ZIF-8, DOX, and DOX@ZIF-8 against HepG-2 and MCF-7 cell lines were evaluated. It was observed that DOX@ZIF-8 had a higher IC_50_ value than DOX; thus, the cytotoxicity of DOX@ZIF-8 was weaker compared to DOX. Although a small amount of DOX was loaded into DOX@ZIF-8, it was observed that the IC_50_ values were close to the IC_50_ values of free DOX in both cell lines. It is also said that these results confirm that a large amount of nanoparticles can be internalized into cancer cells and increase the efficiency of the drug. In addition, it is added that since the pH value of endosomes and lysosomes is acidic, the adopted DOX@ZIF-8 nanoparticles are predicted to release DOX quickly and abundantly into the cell.^42^ In a study in which LAP was encapsulated in lipoprotein-like nanoparticles, it was noted that lipoprotein-like nanoparticles (LTNPs) incorporated with LAP resulted in lower cytotoxicity compared to free LAP (LAP suspension (LTS)) in the BT-474 breast cancer cell line.^62^ These results suggest that considering that only 6.55% of LAP@ZIF-8 used in the experiment was LAP, a high amount of nanoparticles could be taken up by cancer cells, and the efficiency of LAP could be increased. The goal of anticancer drug delivery, such as protection of LAP from plasma proteins and early clearance from the bloodstream,^63^ appears to have been achieved by using ZIF-8 as a nanocarrier in this study. In the study, ZIF-8 nanoparticles, which served as LAP carriers, retained their activity.

The cytotoxic effects of free LAP and LAP@ZIF-8 were evaluated on nontumorigenic MCF-10A mammary epithelial cells to assess their safety profile (Figure 7a–c). Both treatments maintained ≥90% cell viability at concentrations ≤1 μg mL^–1^ across 24, 48, and 72 h, with no statistically significant difference compared to untreated controls (*p* > 0.05). A moderate yet statistically significant reduction in viability (*p* < 0.05) was observed at 10 μg mL^–1^, with survival ranging from 65 to 80%. At the highest concentration tested (100 μg mL^–1^), viability remained above 50%, ranging between 55 and 70%, indicating that the IC_50_ was not reached under these experimental conditions. The observation that MCF-10A cells maintain >50% viability even at 100 μg mL^–1^ is biologically plausible—healthy epithelial cells are typically more resistant to xenobiotic stress than their malignant counterparts—and provides a preliminary safety window for subsequent *in vivo* studies. In contrast, the same concentrations of LAP@ZIF-8 induced significant cytotoxicity in the SKBR-3 and MCF-7 breast cancer cells. These findings demonstrate that LAP@ZIF-8 selectively targets malignant cells while sparing healthy epithelial cells, highlighting its potential as a safe and effective nanocarrier-based anticancer strategy.

#### 3.3.1 Coefficient of Variation for Cell Viability

The coefficient of variation is a dimensionless statistical tool that shows the relationship between the mean and distribution of data and allows variables to be compared independently of scale effects. It is used to express the precision and repeatability of an assay. It is defined as the ratio of the standard deviation to the mean and is often expressed as a percentage. A coefficient of variation above about 30% is considered an indication that there is a problem with the data or that the experiment is out of control.^64^ The coefficient of variation is also known as the relative standard deviation.^65^ The lower the coefficient of variation values, the smaller the spread of results and the higher the precision, while the higher the coefficient of variation values, the larger the spread of results and the lower the precision. When the results were evaluated, it was seen that the coefficient of variation values did not exceed 30% in both cell lines, as shown in the graphs in Figure 8. These results show that there are no problems with the data set or experiment, as well as the consistency, precision, and reproducibility of the data.

**Figure 8:**
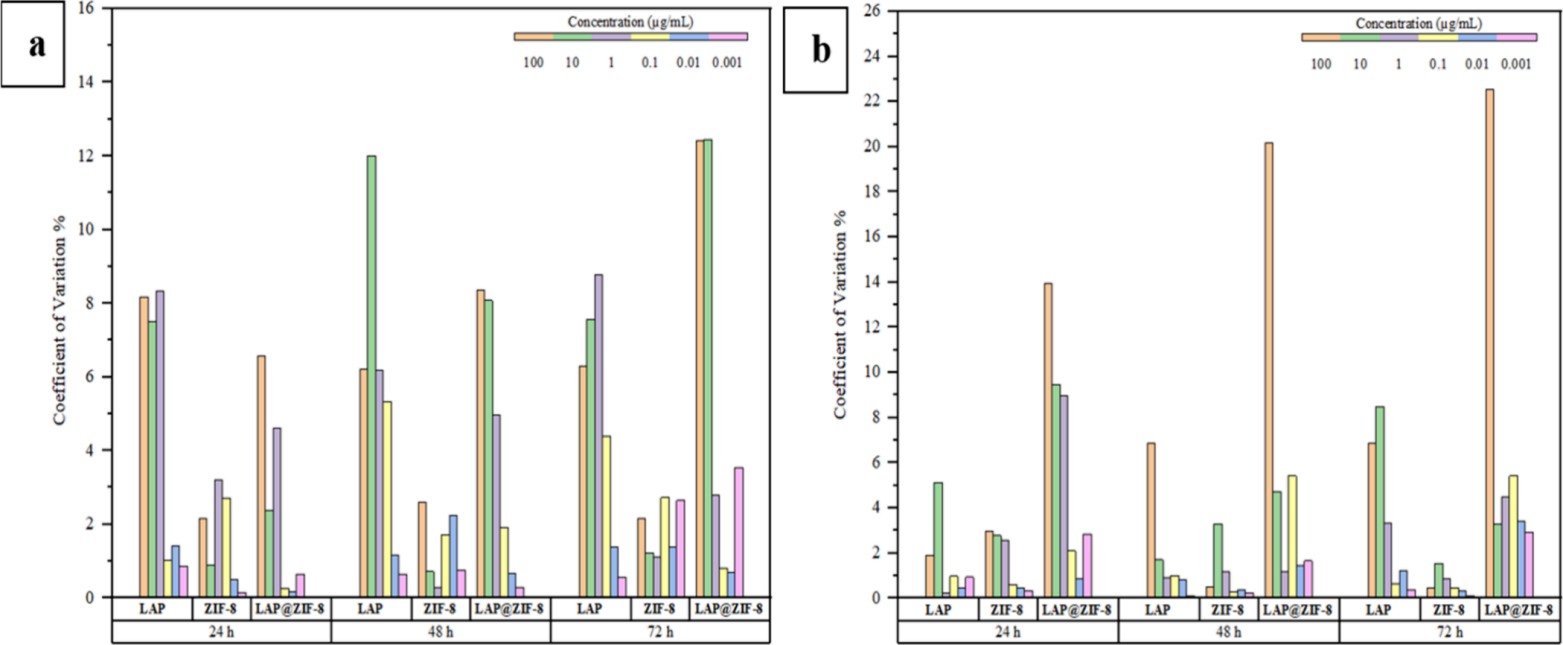
Coefficient of variation of average cell viabilities for different concentrations and incubation times of samples in (a) SKBR-3 and (b) MCF-7 cell lines.

### 3.4 Antibacterial Activity by the Counting-Colony Method

The serial dilution and spread plate technique was used to count viable cells in bacterial solutions. Accordingly, the antibacterial activities of control, LAP, and LAP@ZIF-8 were tested against the Gram-positive bacteria, S. aureus, and against the Gram-negative bacteria, *E. coli* (Table 5 and Figure 9). At the same time, the colonies formed as a result of the treatment of the substances on the bacteria are visualized in Petri dishes. LAP@ZIF-8 exhibited significant antibacterial activity against both of the mentioned strains. The minimum bactericidal concentration (MBC) was determined as the lowest extract concentration killing 99.9% of the bacterial inoculum after 24 h of incubation at 37 °C.^66^ According to the definition and results, LAP@ZIF-8 can be qualified as MBC at a concentration of 5 mg/mL in S. aureus and 10 mg/mL in *E. coli*. The results revealed that LAP@ZIF-8 exhibited superior bacterial cell killing efficacy compared with the free drug formulation. One study found that sorafenib, a tyrosine-kinase inhibitor similar to LAP, had antibacterial activity against S. aureus,^67^ while another study, interestingly, found that LAP had no antibacterial effect on S. aureus at lower concentrations but had effects at higher LAP concentrations.^68^ LAP@ZIF-8 was found to effectively reduce bacterial growth in S. aureus compared to that in *E. coli*, which was thought to be due to the bacterial cell wall difference. The reason for this is that the cell walls of Gram-negative bacteria are more complex (both structurally and chemically). In addition, Gram-positive bacteria have a thin coating of peptidoglycan and teichoic acid in their cell walls that only allows macromolecules to enter, while Gram-negative bacteria have an additional outer membrane consisting of a thick layer of peptidoglycan and lipopolysaccharide that causes intense membrane destruction and cell death.^69^ Antibacterial activities of nanoparticles depend on the physicochemical properties of the nanoparticles and the type of bacteria.^70^ The size, surface area, morphology, and net charge of nanoparticles affect their antibacterial activity. It has been reported that as the size of nanoparticles decreases, their increased surface area provides better interaction with microorganisms, positively charged nanoparticles bind well to negatively charged bacterial surfaces, and the large surface area of spherical nanoparticles allows more ions to be released. All these features have shown that nanoparticles have and will have better antibacterial effects.^71^ The small size, hexagonal spherical morphology, and positive surface charge of LAP@ZIF-8 enabled them to penetrate bacteria more easily and thus exhibit better antibacterial activity than LAP. Since the surface of bacteria usually has a negative charge, positively charged LAP@ZIF-8 nanoparticles are strongly attracted to the bacterial surface via electrostatic interactions, resulting in increased antibacterial activity. In this study, the combination of LAP and ZIF-8, and the amine groups from LAP, which have a bactericidal effect by coordinating to release metal ions (Zn^2+^) that can destroy bacterial cells,^72^ provided an antibacterial ability due to the synergistic effects of these materials. It is worth noting that ZIF-8, as the nanoparticle matrix, may contribute to antibacterial activity through the release of Zn^2+^ions, which are known to disrupt bacterial cell membranes and promote ROS-mediated damage.^73^ In our study, the ZIF-8-only control group showed limited antibacterial activity, supporting this role. However, the significantly enhanced efficacy of LAP@ZIF-8 compared to both free LAP and ZIF-8 alone suggests a synergistic effect between LAP and Zn^2+^. While this supports the dual contribution hypothesis, future studies involving quantitative Zn^2+^release profiles and parallel testing with free Zn^2+^ions would provide a more precise understanding of the individual and combined roles in bacterial inhibition. This study demonstrated the potential of LAP@ZIF-8 to be used as an antibacterial agent and suggested that it can be recommended for biomedical and pharmaceutical applications. To further validate the antibacterial potential of LAP@ZIF-8, future studies should expand the testing panel to include clinically relevant multidrug-resistant strains, anaerobic bacteria, and other pathogenic isolates. This broader-spectrum assessment would provide a more comprehensive understanding of its therapeutic applicability, especially in combating resistant infections.

**Table 5:** Antibacterial Activity of LAP and LAP@ZIF-8 against *S. aureus* and *E. coli*

		reduction in viability (%)	
	sample concentration (mg/mL)	*S. aureus*	*E. coli*
control		0	0
LAP	5	23.81 ± 9.52	24.06 ± 4.06
	10	56.26 ± 2.93	42.10 ± 2.10
LAP@ZIF-8	5	99.91 ± 0.09	65.18 ± 2.68
	10	100	100

**Figure 9:**
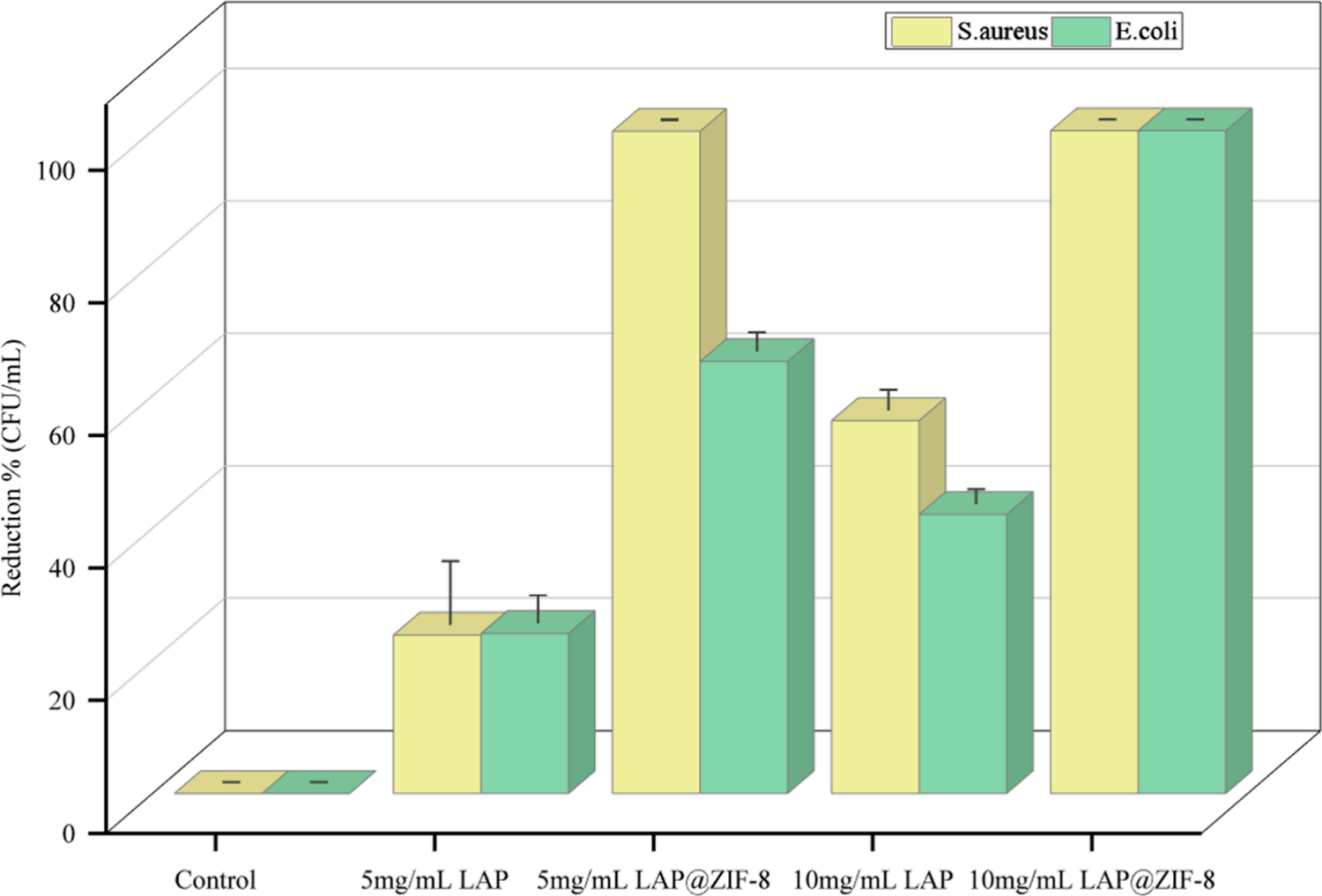
Percent of bacterial reduction (reduction %) of LAP and LAP@ZIF-8 against *S. aureus* and *E. coli*.

### 3.5 Antioxidant Activity by the 1,1-Diphenyl-2-picrylhydrazyl (DPPH) Radical Scavenging Assay

DPPH is a compound that decreases upon exposure to proton radical scavengers, and the DPPH radical scavenging of antioxidants is attributed to the hydrogen donating ability of the substances.^74^ The DPPH radical scavenging assay was performed to evaluate the antioxidant activities of LAP, LAP@ZIF-8, and ascorbic acid. It was found that all of the samples examined had lower radical scavenging activity compared to the antioxidant substance ascorbic acid. The maximum radical scavenging % result was obtained with ascorbic acid as 97.74%, while they were 17.59 and 21.91% for a 200 μg/mL concentration of LAP and LAP@ZIF-8, respectively. As shown in Figure 10, the radical scavenging activity of samples increased with increasing concentration. LAP@ZIF-8 had a better DPPH scavenging activity compared to LAP. The concentration of the antioxidant sample required to scavenge 50% of DPPH radicals is expressed by IC_50_. There is an inverse relationship between the IC_50_ value and DPPH scavenging activity. The lower the IC_50_ value, the stronger the DPPH scavenging activity and antioxidant activity of the sample. IC_50_ values for LAP, LAP@ZIF-8, and ascorbic acid were determined as 803.92, 666, and 9.72 μg/mL, respectively. In the literature, IC_50_ values with ascorbic acid were found as 4.89 μg/mL,^75^ 8.4 μg/mL,^76^ 11.2 μg/mL,^77^ and 28 μg/mL.^78^ The ascorbic acid result obtained in this study is similar to the study conducted by Brighente et al.^77^ Zinc is one of the important minerals with antioxidant properties. It protects cells against oxidative damage and acts as a cofactor for enzymes involved in the proper functioning of the antioxidant defense system.^79^ Zinc has a dual effect in neutralizing free radicals, either directly via glutathione or indirectly as a glutathione peroxidase cofactor. This was reflected in the results, and it was seen that the synthesized nanoparticle was more effective compared to the pure drug. LAP@ZIF-8, which is effective in the treatment of free radical-induced diseases such as cancer, was found to have antioxidant properties. While the DPPH assay provides valuable preliminary data on the free radical scavenging capacity of LAP@ZIF-8, it is an *in vitro* chemical method that does not fully mimic the complex oxidative stress conditions in biological systems. Nevertheless, the observed antioxidant activity suggests the potential for modulating oxidative environments associated with cancer progression. In the context of cancer therapy, where reactive oxygen species (ROS) can contribute to both tumor growth and therapy-induced toxicity, materials with antioxidant potential may help balance ROS levels and protect healthy tissues.^79^ To better assess the *in vivo* relevance, future studies should include cell-based ROS inhibition assays and animal models of oxidative stress, which would provide deeper insights into the biological antioxidant effects of LAP@ZIF-8.

**Figure 10:**
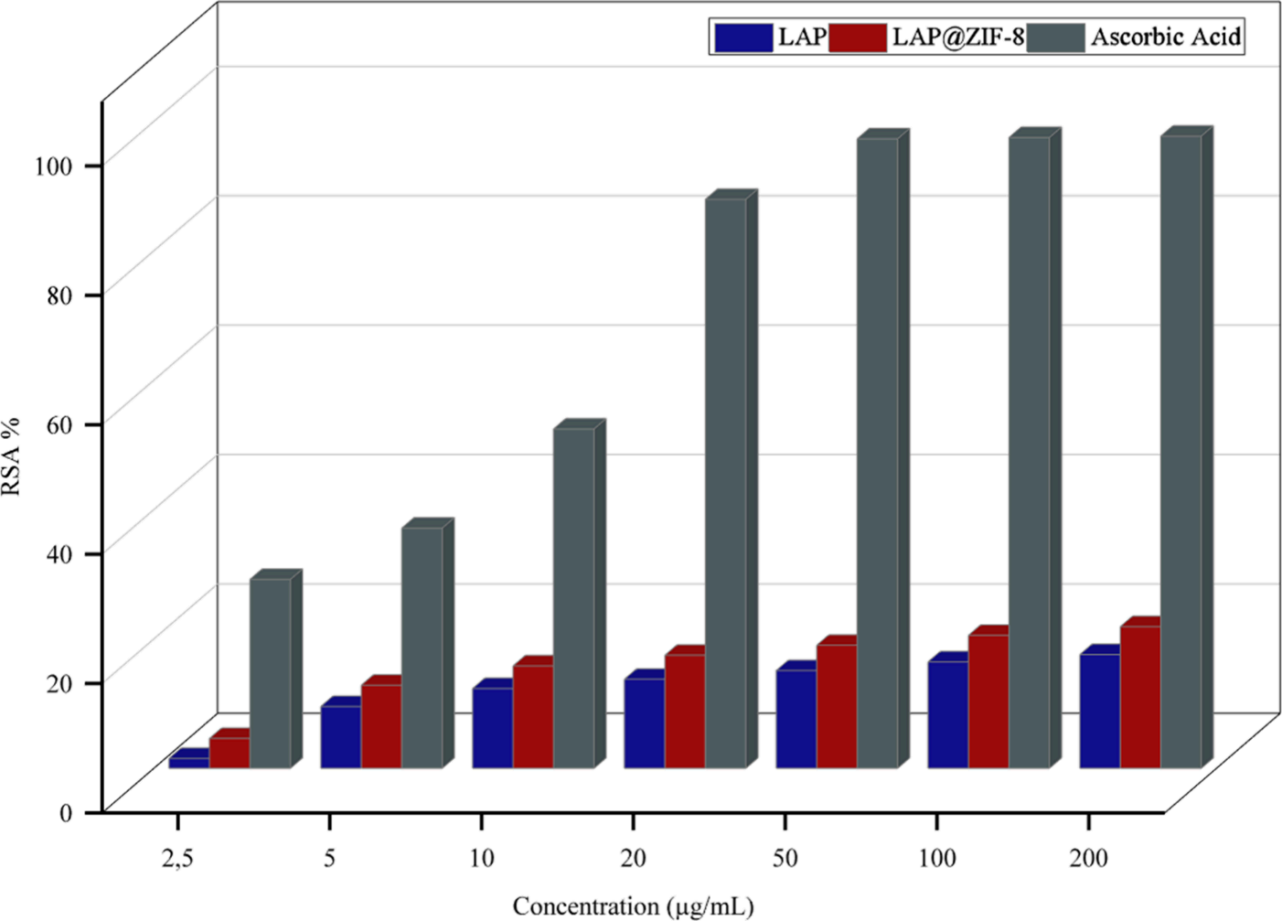
Antioxidant activity (RSA %) of LAP and LAP@ZIF-8 compared to that of ascorbic acid.

## 4 Conclusions

The combination of biocompatibility, cytotoxicity, antibacterial, and antioxidant properties significantly broadens the potential applications of LAP@ZIF-8. These results highlight the material’s potential as a pH-sensitive and targeted drug delivery system for effective cancer treatment, as well as its utility as an antibacterial and antioxidant agent. Given its promising capabilities, LAP@ZIF-8 represents a valuable platform for further development in pharmaceutical applications. Additional comprehensive studies are recommended to elucidate its biological effects and explore its potential use as a drug delivery vehicle in future clinical trials.

LAP@ZIF-8 nanoparticles were successfully synthesized by a one-pot method, achieving an encapsulation efficiency of 72.4% and a drug loading capacity of 6.6%. Detailed physicochemical analyses (SEM, EDX, DLS, ζ-potential, FT-IR, XRD, and TGA) confirmed their uniform morphology, stability, and overall biocompatibility, validating LAP@ZIF-8 as a robust drug delivery platform. Drug release studies revealed a pronounced pH-responsive profile: ∼77% cumulative LAP release at pH 5.5 versus 43% at pH 7.4 over 96 h, an attribute that minimizes premature leakage and favors tumor-selective delivery.

*In vitro* cytotoxicity assays demonstrated that LAP@ZIF-8 matched—and in some cases exceeded—the efficacy of free LAP against SKBR-3 and MCF-7 breast-cancer cells. Crucially, parallel tests on nontumorigenic human mammary epithelial MCF-10A cells showed a distinctly safer profile: cell viability remained ≥90% at ≤1 μg mL^–1^ and exceeded 50% even at 100 μg mL^–1^, indicating that the IC_50_ was not reached and establishing a clear therapeutic window between malignant and healthy cells.

Beyond its anticancer performance, LAP@ZIF-8 exhibited bactericidal activity two- to four-fold greater than LAP alone against both *Escherichia coli* and *Staphylococcus aureus* and displayed a moderate but meaningful DPPH free radical-scavenging capacity. These combined cytotoxic, antibacterial, and antioxidant properties broaden the clinical utility of LAP@ZIF-8 and underscore its multifunctional potential.

Taken together, the favorable biocompatibility in MCF-10A cells, strong anticancer selectivity, and ancillary antibacterial and antioxidant effects position LAP@ZIF-8 as a promising pH-sensitive nanocarrier for targeted breast-cancer therapy. Future work should focus on optimizing its reactive-oxygen-species-scavenging capacity and undertaking comprehensive *in vivo* studies to translate these *in vitro* findings into clinically relevant outcomes.

## Data Availability

All data supporting the findings of this study are available within the manuscript.
